# Real-world outcomes of the CROSS regimen in patients with resectable esophageal or gastro-esophageal junction adenocarcinoma: a nationwide cohort study in the Netherlands

**DOI:** 10.1016/j.eclinm.2024.103067

**Published:** 2025-01-22

**Authors:** Hanneke van Laarhoven, Rob Verhoeven, Mark van Berge Henegouwen, Nadia Haj Mohammad, Richard van Hillegersberg, Marije Slingerland, Christina T. Muijs, Bas Wijnhoven, Bianca Mostert, Laurens Beerepoot, Grard Nieuwenhuijzen, Sarah Derks, Peter S.N. van Rossum

**Affiliations:** a Department of Medical Oncology, Amsterdam UMC Location University of Amsterdam, Meibergdreef 9, Amsterdam, AZ, 1105, the Netherlands; b Cancer Center Amsterdam, Cancer Treatment & Quality of Life, Amsterdam, the Netherlands; c Department of Research & Development, Netherlands Comprehensive Cancer Organization (IKNL), Utrecht, the Netherlands; d Department of Surgery, Amsterdam UMC Location University of Amsterdam, Meibergdreef 9, Amsterdam, AZ, 1105, the Netherlands; e Department of Medical Oncology, University Medical Center Utrecht, Utrecht University, Utrecht, the Netherlands; f Department of Surgery, University Medical Center Utrecht, Utrecht, the Netherlands; g Department of Medical Oncology, Leiden University Medical Center, Leiden, the Netherlands; h Department of Radiation Oncology, University Medical Center Groningen, Groningen, the Netherlands; i Division of Surgical Oncology and Gastrointestinal Surgery, Department of Surgery, Erasmus MC Cancer Institute University Medical Center, Rotterdam, the Netherlands; j Department of Medical Oncology, Erasmus MC Cancer Institute University Medical Center, Rotterdam, the Netherlands; k Department of Medical Oncology, Elisabeth-Tweesteden Hospital, Tilburg, the Netherlands; l Department of Surgery, Catharina Hospital, Eindhoven, Netherlands; m Department of Medical Oncology, Amsterdam UMC Location Free University, Amsterdam, the Netherlands; n Department of Radiation Oncology, Amsterdam UMC Location Free University Amsterdam, Amsterdam, the Netherlands

**Keywords:** Esophageal adenocarcinoma, Neoadjuvant chemoradiotherapy, Pathological complete response, Overall survival, Real-world data

## Abstract

**Background:**

Recent studies in patients with resectable adenocarcinoma of the esophagus or gastroesophageal junction (GEJ)—Neo-AEGIS and ESOPEC—have explored the comparison of neoadjuvant chemoradiotherapy (nCRT) with chemotherapy, with conflicting results. To contextualize the findings from these studies using nCRT as a comparator, we aimed to investigate contemporary real-world outcomes of nCRT in patients with adenocarcinoma of the esophagus or GEJ.

**Methods:**

From the Netherlands Cancer Registry, patients were selected who were diagnosed between 1 January 2015 and 31 December 2022 with a resectable (cT1N+M0 or cT2-4aNanyM0) esophageal, GEJ or gastric cardia adenocarcinoma and started treatment with nCRT according to the CROSS regimen, that is 5 weekly cycles of carboplatin (AUC 2 mg/mL per minute) and paclitaxel (50 mg/m^2^) combined with concurrent radiotherapy (41.4 Gy in 23 fractions of 1.8 Gy). Pathologic complete response (pCR) according to Mandard was the primary outcome of this study and defined as complete tumor regression of the primary tumor (Mandard grade I) irrespective of residual nodal involvement.

**Findings:**

Of the 4765 included patients, 4170 (87.5%) completed the full CROSS regimen of radiotherapy and chemotherapy. A pCR was observed in 704 (20.5%) of 3439 patients who underwent surgical resection within 16 weeks after completing the CROSS regimen. In the complete study population, the median overall survival (OS) was 33.7 months (95% CI 32.0–35.6), with a 3-year OS rate of 48.1%.

**Interpretation:**

Although survival rates in real-world settings are often lower compared to clinical trials, in our real-world cohort the 3-year OS was only 2.6% lower compared to that reported for the group that underwent nCRT in ESOPEC. These real-world results underscore the potential of the CROSS regimen in daily clinical practice.

**Funding:**

None.

**Research in context**

**Evidence before this study**

For the purpose of this study, we conducted a PubMed search for phase III randomized clinical trials up to December 22, 2024, using the keywords “neoadjuvant chemoradiotherapy”, “CROSS”, and “esophageal adenocarcinoma”, without any language restrictions. This search yielded two clinical trials reporting outcomes: the CROSS study, which compared neoadjuvant chemoradiotherapy (nCRT) followed by surgery with surgery alone in patients with resectable esophageal or gastroesophageal junction (GEJ) cancers, demonstrating a survival benefit for nCRT; and the Neo-AEGIS study, which compared nCRT with chemotherapy in patients with resectable esophageal or GEJ adenocarcinoma, indicating no clear superiority between the two treatments. Additionally, the search retrieved the study protocol of ESOPEC, a phase III trial comparing nCRT with peri-operative 5-fluorouracil, leucovorin, oxaliplatin, and docetaxel (FLOT) in patients with resectable esophageal or GEJ adenocarcinoma. The initial results of the ESOPEC trial, presented at ASCO 2024, showed a significant overall survival (OS) benefit favoring chemotherapy.

**Added value of this study**

The efficacy observed in controlled trials does not always translate into a comparable effectiveness in everyday clinical practice. To date, contemporary real-world outcomes of nCRT in patients with adenocarcinoma of the esophagus or GEJ are unavailable. This study investigated all patients from the Netherlands Cancer Registry (NCR)—a nationwide population-based cancer registry that covers the entire Dutch population of approximately 18 million people—who were diagnosed between 1 January 2015 and 31 December 2022 with a resectable (cT1N+M0 or cT2-4aNanyM0) esophageal, GEJ or gastric cardia adenocarcinoma and started treatment with nCRT in a Dutch hospital. An 82% completion rate of the CROSS regimen was observed and a pathologic complete response (pCR) of the primary tumor of nearly 20%. Patients who achieved pCR had a substantially higher OS compared to those with an incomplete response. A median OS of 33.7 and a 3-year OS rate of 48.1% was observed.

**Implications of all the available evidence**

Peri-operative chemotherapy and nCRT are valuable treatment options for patients with resectable esophageal or gastroesophageal junction (GEJ) cancers. Our real-world data highlight the effectiveness and applicability of the CROSS regimen in daily clinical practice. Subgroups of patients may be selected for either perioperative chemotherapy or nCRT, based on their individual treatment preferences. For example, some patients may opt for nCRT due to the potential for organ preservation through active surveillance following the treatment. Future studies will be critical in clarifying the precise roles of FLOT and CROSS in the management of resectable adenocarcinoma of the esophagus or GEJ.

## Introduction

Since the publication of the CROSS trial in 2012,^1^ advancements in survival of patients with resectable esophageal cancer have been limited. The CROSS trial demonstrated superiority of neoadjuvant chemoradiotherapy (nCRT) followed by surgery over surgery alone, with a median overall survival (OS) of 49.4 months in the multimodal treatment arm compared to 24.0 months in the surgery group (hazard ratio [HR] 0.66; 95% confidence interval [CI] 0.495–0.871; p = 0.003).^1^ Specifically for patients with esophageal adenocarcinoma (EAC), superiority was also demonstrated; OS was 43.2 months in the multimodal treatment arm versus 27.1 months in the group of patients who underwent surgery alone (HR 0.73; 95% CI 0.55–0.98; p = 0.038).^2^ In 2021, the Checkmate-577 trial showed a significant improvement in disease-free survival (DFS) for patients with esophageal cancer who had an incomplete pathologic response following nCRT and surgery—a subgroup with a notoriously poor prognosis,^3^ and who were treated with adjuvant nivolumab.^4^ The median disease-free survival was 22.4 months (95% CI 16.6–34.0) in the nivolumab group, compared to 11.0 months in the placebo group, with a HR for disease recurrence or death of 0.69 (96.4% CI 0.56–0.86; p < 0.001).^4^ In the patients with adenocarcinoma specifically, DFS was 19.4 months in the patients treated with adjuvant nivolumab, while it was 11.1 months for the placebo group (HR 0.75; 95% CI 0.59–0.96). Unfortunately, OS data from Checkmate-577 will not be available until later in 2025, but a nationwide real-world matched comparison suggests an improvement in OS.^5^

Recent studies have explored the comparison of neoadjuvant chemoradiotherapy with chemotherapy, based on the hypothesis that the inclusion of radiotherapy for its locoregional effects may be unnecessary in an era of radical en-bloc surgery and lymphadenectomy, while perioperative chemotherapy could offer the potential advantage of reducing the rates of systemic failure. The Neo-AEGIS trial compared trimodality therapy (CROSS regimen) with perioperative chemotherapy regimens (epirubicin plus cisplatin or oxaliplatin plus 5-fluorouracil or capecitabine—a modified MAGIC regimen—before 2018, and 5-fluorouracil, leucovorin, oxaliplatin, and docetaxel (FLOT) thereafter).^6^ To date, Neo-AEGIS provides the largest fully published comprehensive randomized dataset for patients with adenocarcinoma of the esophagus or gastroesophageal junction (GEJ) although the study was prematurely closed with a total of randomized 377 patients, 70% of the target population. It reported a median overall survival of 48.0 months (95% CI 33.6–64.8) in the perioperative chemotherapy group and 49.2 months (34.8–74.4) in the nCRT group (3-year OS 55% versus 57%; HR 1.03; 95% CI 0.77–1.38; p = 0.82), and no major differences in surgery-related complications and health-related quality of life outcomes. In contrast, the German ESOPEC trial, of which the abstract was recently presented at ASCO 2024, comparing peri-operative FLOT to nCRT according to the CROSS regimen for patients with resectable adenocarcinoma of the esophagus or GEJ, reported a median OS for patients treated with FLOT of 66 months, while it was 37 months for patients treated with CROSS, with a 3-year OS of 57.4% versus 50.7%, respectively (HR 0.70; 95% CI 0.53–0.92; p = 0.012).^7^

While the Neo-AEGIS results suggest equipoise, the recent ESOPEC data prompts reconsideration of whether perioperative FLOT should replace CROSS as the current standard multimodal treatment for resectable adenocarcinoma of the esophagus or GEJ. However, although randomized controlled trials provide the most robust evidence for determining optimal treatment regimens, the efficacy observed in controlled trials does not always translate into a comparable effectiveness in everyday clinical practice.^8^^,^^9^ Specifically, a real-world population-based study using data from the Netherlands Cancer Registry (NCR) found no significant overall survival improvement for FLOT-treated gastric cancer patients compared to those receiving anthracycline triplets.^10^ However, FLOT-treated patients did show higher rates of completing neoadjuvant therapy, proceeding to adjuvant therapy, and achieving pathologic complete response (pCR).^10^ This discrepancy between selected patients included in trials and the entire (unselected) patient population underscores the need to bridge the gap between clinical trial outcomes and real-world application. To contextualize the findings from the recent randomized studies using nCRT as a comparator, we aimed to investigate the unselected contemporary real-world outcomes of nCRT in patients with adenocarcinoma of the esophagus or GEJ. Our primary focus was on pathological response, while OS served as a key secondary endpoint.

## Methods

### Study design

The Netherlands Cancer Registry (NCR) is a nationwide population-based cancer registry that covers the entire Dutch population of approximately 18 million people. The NCR is based on notifications of all newly diagnosed malignancies in the Netherlands, and trained NCR employees routinely extract information on diagnosis, tumor stage, treatment and outcomes from medical records and register according to a standardized coding manual. In the NCR, sex is classified at birth as male, female or hermaphrodite. In consideration of the absence of any data on the gender of the included patients, we use the terms sex and male/female when referring to the study participants. As the study is based on patients treated in daily clinical practice in all Dutch hospitals, diagnostic procedures are not standardized for this specific study. Tumor location and N staging will primarily be determined based on pre-therapeutic information. The multidisciplinary tumor board's conclusion, if clearly stated, will generally serve as the definitive source for tumor location. In the absence of such a conclusion, location will be established using endoscopic findings. If endoscopic data is unavailable or inconclusive, computed tomography (CT) imaging will be utilized to ascertain the tumor's location. Tumor topography and morphology are coded according to the International Classification of Diseases for Oncology (ICD-O). Information on vital status is obtained through annual linkage with the Municipal Personal Records Database and has been completed until January 31st 2024.

### Ethics

This study did not need approval by an Institutional Review Board in the Netherlands according to the Central Committee on Research Involving Human Subjects. In the context of the NCR, informed consent from patients is not required for the analysis of their data due to specific legal exemptions and derogations outlined in the EU's General Data Protection Regulation (GDPR) and national legislation. This study was approved by the Privacy Review Board of the NCR (request number 24-00312) and the scientific committee of the Dutch Upper GI Cancer Group and follows the Strengthening the Reporting of Observational Studies in Epidemiology (STROBE) statement.^11^

### Study population

**Fig. 1:**
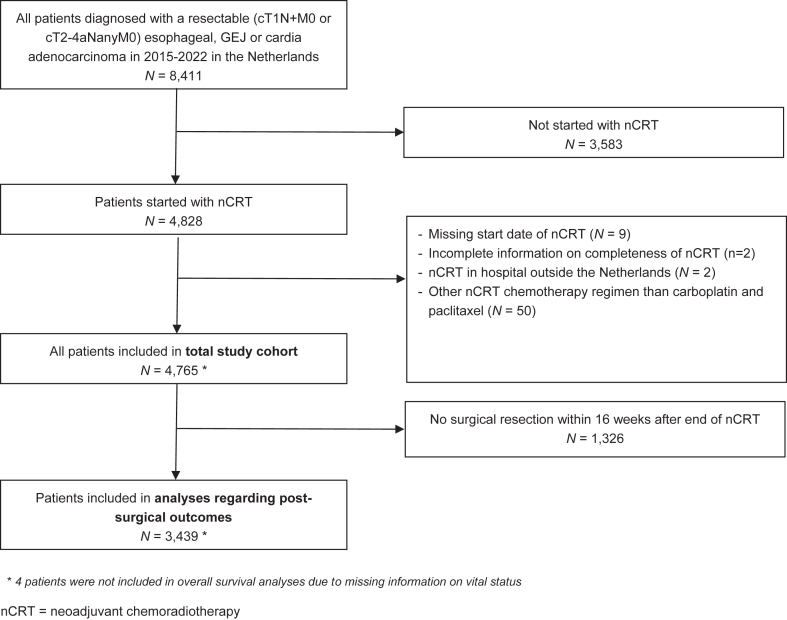
*Flowchart on the inclusion and exclusion of patients into the current study.* ∗*4 patients were not included in overall survival analyses due to missing information on vital status*. nCRT = neoadjuvant chemoradiotherapy.

From the NCR, all patients were selected who were diagnosed from 1 January 2015 until 31 December 2022 with a resectable (cT1N+M0 or cT2-4aNanyM0) esophageal, GEJ or gastric cardia adenocarcinoma and started treatment with nCRT in a Dutch hospital (Fig. 1). Although all patients were deemed clinically resectable, a significant proportion did not undergo surgery following nCRT. This may be attributed to the SANO trial, which enrolled patients in the study population between 8 November 2017 and 17 January 2021, offering those who achieved a clinical complete response to enter an active surveillance protocol.^12^ During the first 4.5 months of the trial, all centers provided standard immediate surgery. Only after 4.5 months, a cluster of two started to provide the novel strategy (active surveillance).^13^ Alternatively, other factors such as disease progression on ^18^F-FDG PET-CT before surgery—standard practice in the Netherlands—or deterioration in performance status may have contributed to the decision to omit surgery. After the SANO trial, 11 centers continued the active surveillance approach.^14^ Since in all the patients in whom surgical resection was omitted pathologic responses could not be evaluated, post-surgical outcomes including pathologic response were only determined in the subgroup of patients who underwent surgical resection within 16 weeks after the end of nCRT.

### Neoadjuvant chemoradiotherapy

According to the Dutch guidelines, the recommended regimen for nCRT is based on the CROSS trial, which includes 5 weekly cycles of carboplatin (AUC 2 mg/mL per min) and paclitaxel (50 mg/m^2^) combined with concurrent radiotherapy (41.4 Gy in 23 fractions of 1.8 Gy). For this study, we included all patients who began chemoradiotherapy with neoadjuvant intent, defined as receiving at least 1 cycle of carboplatin and paclitaxel and at least 1 fraction of external radiotherapy, regardless of whether they proceeded to surgery. To exclude patients who may have been treated with non-surgical definitive CRT intent (with generally a radiotherapy dose of 50–50.4 Gy), we excluded those who received a radiotherapy dose greater than 41.4 Gy but did not undergo surgery.

### Definitions

The neoadjuvant radiotherapy component of the nCRT protocol was considered complete if a dosage of ≥41.4 Gy was administered or—in case the dosage was missing—a minimum radiotherapy duration of 30 days. The neoadjuvant chemotherapy component was deemed complete if at least 5 cycles of both carboplatin and paclitaxel were delivered or—in case the number of cycles was missing—a minimum duration between first and last chemotherapy administration of 28 days. The entire nCRT regimen was considered complete if both the neoadjuvant radiotherapy and chemotherapy components met these criteria.

pCR according to Mandard was the primary outcome of this study and defined as complete tumor regression of the primary tumor (Mandard grade I) irrespective of residual nodal involvement.^15^ All patients with a known but incomplete tumor regression were classified as ‘No pCR’. In addition, we presented pathologic response according to the ypTNM stage. Herein, any presence of residual invasive tumor at the primary site (ypT1–4) was classified as ypT+, and residual involvement of lymph nodes was classified as ypN+ (ypN1–3). Subsequently patients were grouped into: ypT0N0, ypT+N0, ypT0N+, ypT+N+, ypM1, or ypTNM unknown. The secondary outcome OS was defined as the interval between start of nCRT and death or last follow-up. For some additional analyses based on surgical patients only, OS was defined as the interval between surgical resection and death or last follow-up.

### Statistical analysis

The frequency and proportion of missing data per variable is presented in Supplementary Table S1. To assess the missingness of data, the association of being missing of a certain variable with other variables in the dataset was assessed with both the missing_pairs and missing_compare function of the R package called finalfit. The missing_pairs function was used to produce a visual overview on the association of the missingness of a variable with all other variables. The missing_compare function was used to test the association of the missingness of each variable with other variables (all output included in Supplementary File Missing Data). Except for cN-stage, all variables had a significant association with one or multiple other variables in the dataset. Therefore, the values of missing data were not considered ‘missing completely at random’, but as ‘missing (conditionally) at random’. Missing data of these variables were thereafter imputed using random forest imputation from the R-package missRanger. As missing values of cN-stage might be ‘missing completely at random’ the missing values of this variable were not imputed, but were reported as missing data in Table 1.

**Table 1:** Patient, tumor, and treatment characteristics of all patients who started with neoadjuvant chemoradiotherapy (nCRT).

	N	%
**Age groups**		
18–49	195	4.1%
50–64	1704	35.8%
65–79	2709	56.9%
≥80	157	3.3%
**Sex**		
Male	4003	84.0%
Female	762	16.0%
**Number of comorbidities**		
No comorbidities	2571	54.0%
1 comorbidity	1472	30.9%
≥2 comorbidities	722	15.2%
**WHO performance status at diagnosis**		
0	2827	59.3%
1	1795	37.7%
≥2	143	3.0%
**Year of diagnosis**		
2015	604	12.7%
2016	617	13.0%
2017	634	13.3%
2018	587	12.3%
2019	612	12.8%
2020	546	11.5%
2021	609	12.8%
2022	556	11.7%
**Primary tumor location**		
Upper & middle third of esophagus	130	2.7%
Distal third of esophagus	4098	85.9%
Gastro-esophageal junction & cardia	525	11.0%
Overlapping parts esophagus	18	0.4%
**Tumor differentiation grade**		
Well and moderately differentiated	2654	55.7%
Poorly and undifferentiated	2111	44.3%
**cT stage**		
cT1	14	0.3%
cT2	1327	27.9%
cT3	3369	70.7%
cT4	55	1.2%
**cN stage**		
cN0	1935	40.6%
cN+	2795	58.7%
cNX	35	0.7%
**Lauren type**		
Intestinal	3623	76.0%
Diffuse	808	17.0%
Mixed	127	2.7%
Indeterminate	207	4.3%
**Endoscopic ultra sound prior to start of treatment**		
No	2057	43.2%
Yes	2708	56.8%
**PET-(CT) scan prior to start of treatment**		
No	222	4.7%
Yes	4443	95.4%
**Dose reduction of neoadjuvant chemotherapy** (only available for patients since 2018)		
No dose reduction	2759	94.8%
Dose reduction	151	5.2%
**Type of surgical resection**		
No resection	992	20.8%
Esophagectomy	3728	78.2%
Total gastrectomy	45	0.9%
**Adjuvant treatment**		
No adjuvant nivolumab	3428	71.9%
Adjuvant nivolumab	345	7.2%
Not applicable, no surgical resection	992	20.8%

To assess the impact of pCR on OS, we analyzed OS based on pathologic response in patients who underwent surgical resection within 16 weeks after completing nCRT. OS was presented using Kaplan–Meier curves, with differences in OS assessed using the log-rank test (based on PROC LIFETEST procedure in SAS). A p-value of <0.05 was considered statistically significant. Except for the random forest imputation which was performed in R Version 4.4.1 using RStudio, all statistical analyses were performed in SAS for Windows version 9.4.

### Sensitivity analyses

A significant proportion of Dutch patients were included in the SANO study and, therefore, might have been placed on the SANO surveillance protocol if they achieved a clinical complete response.^12^ Since pCR can only be confirmed in patients who undergo resection, sensitivity analyses were conducted on two specific cohorts that both lacked interference of the SANO study: the ‘Prior-to-SANO cohort’, which included patients diagnosed before the start of the active surveillance strategy of the SANO trial (i.e., diagnosed from 1 January 2015 until 31 December 2017), and the ‘Non-SANO centers cohort’, which included patients treated at centers that did not participate in the SANO trial (diagnosed in from 1 January 2015 until 31 December 2022). These analyses were aimed at evaluating the potential impact of an active surveillance strategy on the reported pathologic complete response rate. Comparisons between the Prior-to-SANO cohort and total cohort and the Non-SANO center cohort and the total cohort were performed with chi-square analyses.

### Role of a funding source

There was no role of a funding source in the study design, in the collection, analysis, and interpretation of data, in the writing of the report, and in the decision to submit the paper for publication.

## Results

From the NCR, we included 4765 patients with a resectable adenocarcinoma of the esophagus, GEJ or cardia and treated with nCRT according to the CROSS regimen (Fig. 1). Among included patients 4003 (84%) were male and 762 (16%) female (Table 1). The median age of the patients was 67 years (inter-quartile range: 61–72 years). Most patients had a relatively good performance status before the start of nCRT, with 97% classified as WHO 0–1, and the majority had either no comorbidities (59%) or one comorbidity (38%). The majority had a cT3 tumor (n = 3369, 71%) and nodal involvement (cN+, 59%). Of the total 4765 patients who started with nCRT, 3773 (79.2%) underwent a surgical resection. Most frequent reasons for not undergoing a surgical resection were disease progression (41%) or entering an active surveillance protocol, most likely within the SANO study (37%; Supplementary Table S3).

Of all included patients, 4170 (88%) completed the full CROSS regimen of radiotherapy and chemotherapy. Radiotherapy was completed by 4668 patients (98%). Chemotherapy was completed by 4234 patients (89%) (Table 2). In both sensitivity cohorts (Prior-to-SANO cohort and Non-SANO centers cohort) the completion rates of the full CROSS regimen, as well as for radiotherapy and chemotherapy, had a maximum difference of 2.8% (p < 0.01, Table 2).

**Table 2:** Neoadjuvant chemoradiotherapy (nCRT) completion rates of all patients who started with nCRT.

	Total cohort (n = 4765)		Prior-to-SANO cohort (n = 1855)			Non-SANO centers cohort (n = 1255)		
	N	%	N	%	P-value^a^	N	%	P-value^a^
**Neoadjuvant radiotherapy completion rate**					0.07			0.91
Complete	4668	98.0%	1802	97.1%		1229	97.9%	
Incomplete	97	2.0%	53	2.9%		26	2.1%	
**Neoadjuvant chemotherapy completion rate**					0.94			0.01
Complete	4234	88.9%	1647	88.8%		1082	86.2%	
Incomplete	531	11.1%	208	11.2%		173	13.8%	
**Neoadjuvant chemoradiotherapy completion rate**					0.50			<0.01
Complete	4170	87.5%	1612	86.9%		1063	84.7%	
Incomplete	595	12.5%	243	13.1%		192	15.3%	

a Comparison of sub-cohort with total cohort based on Chi-Square test.

A pCR of the primary tumor (according to Mandard) was observed in 704 (20.5%) of 3439 patients who underwent surgical resection within 16 weeks after completing the CROSS regimen (Table 3). The pCR rate of the primary tumor was 20.8% in the Prior-to-SANO cohort sensitivity analysis (p = 0.78) and 21.5% in the Non-SANO Centers cohort (p = 0.48). The proportion of patients with ypT0N0 stage was 18.0%, with similar rates observed in the Prior-to-SANO and Non-SANO Centers cohorts, at 18.2% (p = 0.84) and 19.2% (p = 0.89), respectively. Radical (R0) resection was achieved in 92% of patients, with post-operative mortality rates of 1.9% at 30 days and 4.5% at 90 days.

**Table 3:** Post-surgical outcomes of patients who underwent surgical resection within 16 weeks after the end of neoadjuvant chemoradiotherapy (nCRT).

	Total cohort (n = 3439)		Prior-to-SANO cohort (n = 1547)			Non-SANO centers cohort (n = 991)		
	N	%	N	%	P-value^a^	N	%	P-value^a^
***Tumor regression grade primary tumor***					0.78			0.48
Pathological complete response	704	20.5%	322	20.8%		213	21.5%	
No pathological complete response	2735	79.5%	1225	79.2%		778	78.5%	
***ypTNM stage***					0.84			0.89
ypT0N0	619	18.0%	282	18.2%		190	19.2%	
ypT+N0	1379	40.1%	638	41.2%		402	40.6%	
ypT0N+	85	2.5%	42	2.7%		23	2.3%	
ypT+N+	1309	38.1%	563	36.4%		363	36.6%	
ypM1	47	1.4%	22	1.4%		13	1.3%	
**Surgical radicality**					<0.01			<0.01
Radical (R0)	3149	91.6%	1459	94.3%		871	87.9%	
Irradical (R1/R2)	290	8.4%	88	5.7%		120	12.1%	
**30-day post-operative mortality**					0.79			0.43
Deceased within 30 days post-operative	65	1.9%	31	2.0%		15	1.5%	
Alive 30 days post-operative	3374	98.1%	1516	98.0%		976	98.5%	
**90-day post-operative mortality**					0.82			0.10
Deceased within 90 days post-operative	156	4.5%	68	4.4%		33	3.3%	
Alive 90 days post-operative	3283	95.5%	1479	95.6%		958	96.7%	

a Comparison of sub-cohort with total cohort based on Chi-Square test.

**Fig. 2:**
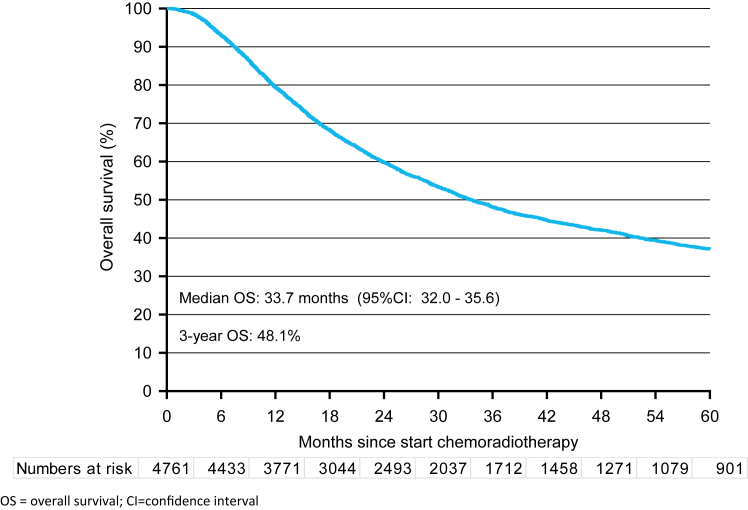
*Overall survival (OS) of total cohort since start of neoadjuvant chemoradiotherapy.* OS = overall survival; CI = confidence interval.

**Fig. 3:**
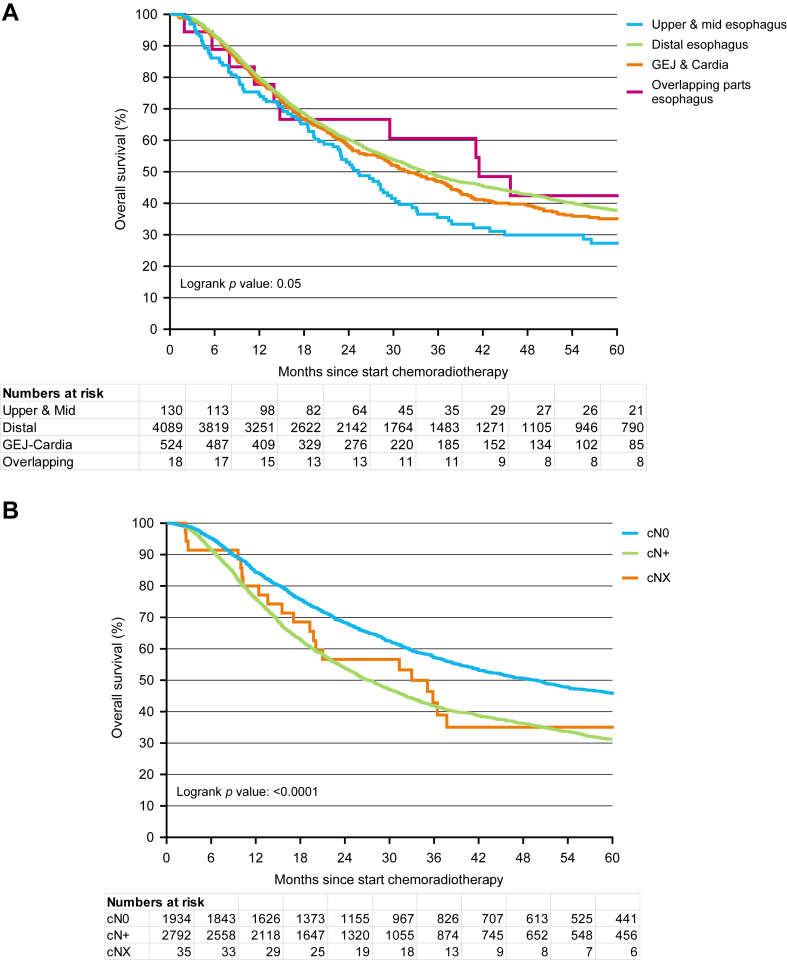
*Overall survival (OS) of total cohort according to primary tumor location (3A) and cN stage (3B)*.

Included patients had a median follow-up time of 25 months and 2771 (58%) patients deceased during the follow-up time period. Only 8 (0.17%) patients were lost to follow-up, which was most likely due to emigration out of the Netherlands. In the complete study population, the median OS was 33.7 months (95% CI 32.0–35.6), with a 3-year OS rate of 48.1% (Fig. 2). The OS was nearly identical in both the Prior-to-SANO cohort (median OS 33.2 months; 3-year OS 48.1%, p-value = 0.55) and the Non-SANO Centers cohort (median OS 33.0 months; 3-year OS 47.8%, p-value = 0.79; Supplementary Figure S1). The location of the primary tumor was not associated with OS (p = 0.05, Fig. 3A). However, the cN-stage prior to treatment was significantly associated with OS, with a 3-year OS rate of 57.3% for cN0 and 41.8% for cN+ (p < 0.0001, Fig. 3B).

**Fig. 4:**
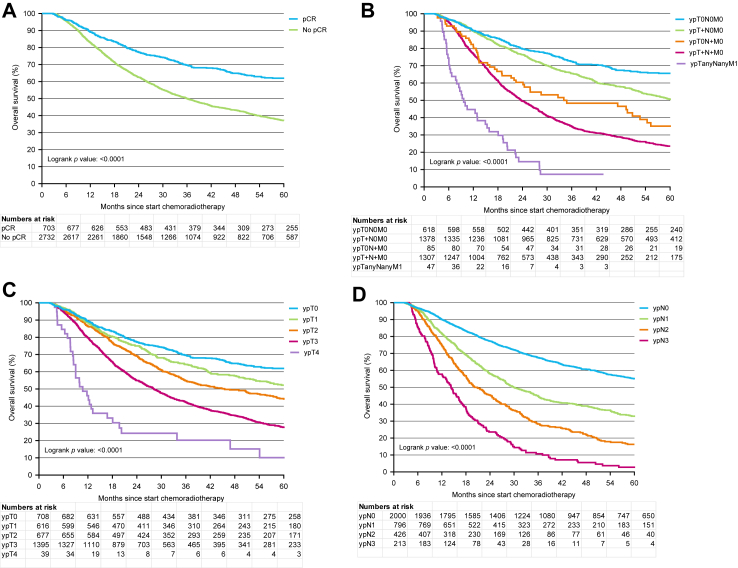
*Overall survival (OS) since start of neoadjuvant chemoradiotherapy (nCRT) of patients who underwent surgical resection within 16 weeks after end of nCRT according to Tumor regression grade primary tumor (4A), ypTNM stage (4B), ypT stage (4C), and ypN stage (4D)*.

Patients with pCR of the primary tumor had significantly better OS compared to those with an incomplete pathologic response, with 3-year OS rates of 69.6% versus 50.1% (p < 0.0001; Fig. 4A). Among the subgroups, patients classified as ypM1 had the lowest 3-year OS of 7.3%, followed by those categorized as ypT+N+ (3-year OS 35.0%), ypT0N+ (48.4%), ypT+N0 (65.5%), and ypT0N0 (72.5%) (p < 0.001; Fig. 4B). Both ypT (Fig. 4C) and ypN (Fig. 4D) stages were also strongly associated with OS (both p < 0.0001).

## Discussion

In this real-world cohort study of patients diagnosed between 1 January 2015 and 31 December 2022—approximately covering the inclusion period of the recently reported Neo-AEGIS and ESOPEC studies—we demonstrated that at least 88% of patients successfully completed the nCRT part of the CROSS regimen. In the original CROSS study 94.7% of the per-protocol population received the full treatment regimen of 5 cycles of chemoradiotherapy.^1^ Similarly, in the trimodality therapy group of the Neo-AEGIS trial 87% of patients completed the full neoadjuvant treatment regime,^6^ but this per-protocol completion rate was only 75% in the ESOPEC study.^7^ A 87.5%, completion rate in a real-world cohort is notably high, especially in contrast to the lower completion rate reported in the ESOPEC study. It may be hypothesized that the long-standing experience with chemoradiotherapy in the Netherlands has contributed to higher completion rates, possibly through more familiarity with the protocol and management of treatment-related side effects. For example, the CROSS protocol allows for continuation of chemotherapy with thrombocytes of ≥50 × 10^9^/L, while many treating physicians may be inclined to withhold chemotherapy when thrombocytes are below 100 × 10^9^/L.

The 3-year OS rates observed in our real-world cohort were only marginally lower (<3%) compared to those reported for groups that underwent nCRT in the Neo-AEGIS and ESOPEC trials, although it is important to highlight that the CROSS group of ESOPEC had 81.6% patients who were cN+ and 81.9% cT3-T4, whereas the corresponding numbers in the current analysis in patients treated with CROSS were 58.7% and 71.9%, respectively. Nevertheless, it is also important to note that survival rates in real-world settings are often lower compared to clinical trials, primarily due to the strict inclusion and exclusion criteria in the latter.^16^ As previously reported,^3^ OS was significantly influenced by pCR. Patients who achieved pCR had a substantially higher OS compared to those with an incomplete response. Notably, the pCR observed in our real-world cohort (approximately 20% overall) aligns closely with the pCR reported for adenocarcinoma patients in the CROSS study (23%),^1^ whereas Neo-AEGIS and ESOPEC reported lower pCR rates of 13.8% and 13.3%, respectively. The reasons for this discrepancy remain unclear, but it could be speculated that systematic differences in the pathological assessment of pCR rates between the UK, Germany and the Netherlands could play a role. Alternatively, tumor-intrinsic differences in sensitivity to chemoradiotherapy across different countries may contribute to the variation observed. The notably high incidence of esophageal adenocarcinoma in the Netherlands compared to other countries^17^^,^^18^ may indicate geographic variations in the genetic or molecular characteristics of tumors, which could also impact treatment responsiveness. Of note, however, in a comparison between Dutch and East Asian patients with esophageal squamous cell carcinoma, significantly higher rates of pathologic complete response were also observed following neoadjuvant chemoradiation.^19^ Further research into these intrinsic tumor characteristics, as well as an analysis of treatment protocols and patient demographics, is needed to clarify the factors driving these variations in treatment response.

Although the reported survival benefit of FLOT over CROSS reported by ESOPEC is compelling, it is important to recognize that conference data are still preliminary. Caution is warranted to avoid prematurely discarding valuable aspects of current practice, as illustrated by the results from our real-world cohort. Several critical pieces of information relevant to treatment decision-making are still unknown for ESOPEC as we await the full publication. For example, data on primary tumor location (GEJ tumors versus tumors distal or more proximal in the esophagus) and nodal status (cN0 versus cN1, cN2, and cN3) in relation to treatment outcomes, as well as treatment-related adverse events have not yet been reported. The latter is particularly important in the context of shared decision-making with patients, as neuropathy—probably more common in patients treated with FLOT—can significantly affect long-term quality of life.^20^ Furthermore, the quality of the radiotherapy, including radiation fields and constraints used for organs-at-risk in the ESOPEC study, which directly impact the efficacy and safety of radiotherapy,^21^ are eagerly awaited in the full publication of the study.

Some limitations of our study need to be mentioned. First, this real-world data study was conducted in the Netherlands and results may not necessarily extrapolate to other parts of the world. Another important limitation is that, in the final years of the study, a significant proportion of patients with a clinically favorable response to nCRT underwent active surveillance. This might have influenced pathologic response rates and OS. However, sensitivity analyses indicated that the outcomes remained relatively consistent, suggesting that the SANO study has not substantially impacted the results of the current study, even though it should be noted that in the Non-SANO centers cohort still some patients may have undergone active surveillance. Importantly, in the SANO study, 2-year OS was shown to be non-inferior with active surveillance compared to standard resection.^12^ Finally, we wish to emphasize that our study does not include a comparison group for perioperative chemotherapy, and in this study we did not aim to compare the efficacy of neoadjuvant chemoradiotherapy with perioperative chemotherapy. Instead, our goal is to present the real-world outcomes of neoadjuvant chemoradiotherapy using the CROSS protocol. A significant strength of this study is its population-based, nationwide design and the large number of included patients. A significant strength of this study is its population-based, nationwide design and the large number of included patients. This approach is particularly important in addressing the efficacy-effectiveness gap, where the outcomes observed in clinical trials may not fully translate to real-world settings.^16^^,^^22^, ^23^, ^24^, ^25^ This gap arises due to differences in patient characteristics, healthcare system factors, and the methodological constraints of RCTs. We believe that both RCTs and real-world studies have their own strengths and contributions to the field, and our study is intended to add to this body of knowledge.

In conclusion, given our real-world results, we recommend exercising caution before discontinuing the use of the CROSS regimen for all individual patients in clinical practice. The potential for this regimen to be effectively combined with targeted therapies,^26^ which may present challenges when used alongside FLOT,^27^ could also justify its continued application. Finally, chemoradiotherapy—but not FLOT—is an established definitive trial-investigated treatment option in the Netherlands in which promising results have been achieved recently with an active surveillance strategy after treatment, yielding organ-preservation in selected patients.^12^ Future studies will be critical in clarifying the precise roles of FLOT and CROSS in the management of resectable adenocarcinoma of the esophagus or GEJ.

## Contributors

Hanneke van Laarhoven, Rob Verhoeven, Mark van Berge Henegouwen, Nadia Haj Mohammad, Richard van Hillegersberg, Marije Slingerland, Christina T. Muijs, Bas Wijnhoven, Bianca Mostert, Laurens Beerepoot, Grard Nieuwenhuijzen, Sarah Derks, and Peter S.N. van Rossum contributed to the conceptualization of the study.

Rob Verhoeven curated the data.

Rob Verhoeven performed the formal data analysis and Rob Verhoeven, Peter van Rossum, and Hanneke van Laarhoven have verified the underlying data.

Rob Verhoeven and Peter S.N. van Rossum developed the methodology.

Rob Verhoeven was responsible for data visualisation.

Hanneke van Laarhoven, Rob Verhoeven, Mark van Berge Henegouwen, Nadia Haj Mohammad, Richard van Hillegersberg, Marije Slingerland, Christina T. Muijs, Bas Wijnhoven, Bianca Mostert, Laurens Beerepoot, Grard Nieuwenhuijzen, Sarah Derks, and Peter S.N. van Rossum contributed to data interpretation.

Hanneke van Laarhoven wrote the original draft.

Hanneke van Laarhoven, Rob Verhoeven, Mark van Berge Henegouwen, Nadia Haj Mohammad, Richard van Hillegersberg, Marije Slingerland, Christina T. Muijs, Bas Wijnhoven, Bianca Mostert, Laurens Beerepoot, Grard Nieuwenhuijzen, Sarah Derks, and Peter S.N. van Rossum contributed to the writing by reviewing and editing.

All authors read and approved the final version of the manuscript.

## Data sharing statement

Data of the Netherlands Cancer Registry (NCR) as used for this study can be made available for scientific purposes based on reasonable request according to standard regulations for data of the NCR via: https://iknl.nl/en/ncr/apply-for-data.

## Declaration of interests

Hanneke van Laarhoven has acted as a consultant or in an advisory role for Amphera, Astellas, Beigene, Daiichy, Myeloid; and has received research funding and/or medication/material supply from AMGEN, AstraZeneca, AURISTONE, BMS, Incyte, Merck, ORCA, and Servier; and speaker roles for Astellas, AstraZeneca, BMS, Benecke, Daiichi-Sankyo, JAAP, Medtalks, Novartis, Servier, and Travel Congress Management; travel support from AstraZeneca; she is chair of the ESMO upper GI faculty.

Rob Verhoeven has received research funding from BMS and Amgen and has acted as consultant for Daiichi Sankyo.

Mark van Berge Henegouwen reports consulting or advisory roles for Viatris, Johnson & Johnson, BBraun, Stryker and Medtronic.

Nadia Haj Mohammad has acted as a consultant or in an advisory role for BMS, Astra Zeneca, Servier, MSD, and Eli Lilly; and has received research funding and/or medication/material supply from Servier.

Richard van Hillegersberg is Procotor for Intutive Surgical, member of advisory board of Medtronic, Olympus and Ethicon.

Marije Slingerland has acted as a consultant or advisory role for BMS, Astra Zeneca and Lilly.

Christina Muijs has no personal declarations of interest.

Bianca Mostert has acted as a consultant or advisory role for BMS, Lilly, Servier, Amgen and AstraZeneca, and has received research funding and/or medication/material supply from BMS and Pfizer.

Bas Wijnhoven has received research funding from BMS and has acted as consultant for Medtronic.

Laurens Beerepoot has had speaker roles for Servier, BMS, Congress Care, Ipsen, Medtalks, Benecke and Travel Congress Management.

Grard Nieuwenhuijzen has acted as a consultant or advisory role for Medtronic.

Sarah Derks has acted as a consultant or advisory role for BMS, has received research funding and/or medication/material supply from Incyte; and speaker roles for BMS, Benecke and Servier.

Peter van Rossum has no personal declarations of interest.

## Appendix ASupplementary data

Supplementary Data

Supplementary Figure S1 Overall survival (OS) of pre-SANO cohort (1A) and Not SANO centers since start of neoadjuvant chemoradiotherapy.
