# Supplementary material for: Real-world outcomes of the CROSS regimen in patients with resectable esophageal or gastro-esophageal junction adenocarcinoma: a nationwide cohort study in the Netherlands
Source: eClinicalMedicine. 2025 Jan 22;80:103067. doi: 10.1016/j.eclinm.2024.103067 (PMC11795631; doi:10.1016/j.eclinm.2024.103067)
Supplement: Supplementary Tables S1–S3 [file mmc1.pdf]

Supplementary tables

Supplementary Table S1. Number and percentage of missing per variable

	N missing	% missing
Age	0	0%
Sex	0	0%
Number of comorbidities	165	3.5%
WHO performance status at diagnosis	420	8.8%
Year of diagnosis	0	0%
Primary tumor location	51	1.1%
Tumor differentiation grade	790	16.6%
cT stage	0	0%
cN stage	35	0.7%
Lauren type	879	18.4%
Endoscopic ultra sound prior to start of treatment	27	0.6%
PET-(CT) scan prior to start of treatment	94	2.0%
Dose reduction of neoadjuvant chemotherapy (*for patients since 2018*)	150	3.1%
Type of surgical resection	22	0.5%
Adjuvant treatment	0	0%
Neoadjuvant radiotherapy completion rate	0	0%
Neoadjuvant chemotherapy completion rate	0	0%
Neoadjuvant chemoradiotherapy completion rate	0	0%
Tumor regression grade primary tumor	245	5.1%
ypT	34	0.7%
ypN	11	0.2%
ypTNM stage	35	0.7%
Surgical radicality	183	3.8%
30-day post-operative mortality	5	0.1%
90-day post-operative mortality	6	0.1%
Reasons for not undergoing a surgical resection of primary tumor after nCRT	115	2.4%
Time between nCRT and resection (in weeks)	3	0.1%
Vital status at end of follow-up	4	0.1%
Follow-up duration	4	0.1%

nCRT – neoadjuvant chemoradiotherapy

Supplementary Table S2. Overview of variable names*

**Variable name**	**Description**
leeft	Age
gesl	Sex
cci_cat	Number of comorbidities
perf_stat	WHO performance status at diagnosis
incjr	Year of diagnosis
topo_sublok	Primary tumor location
diffgrad	Tumor differentiation grade
ct	cT stage
cn2	cN stage
lauren	Lauren type
eus	Endoscopic ultra sound prior to start of treatment
pet_scan	PET-(CT) scan prior to start of treatment
DR	Dose reduction of neoadjuvant chemotherapy (*for patients since 2018*)
chir_type1	Type of surgical resection
adj_nivo	Adjuvant treatment
RT_compleet	Neoadjuvant radiotherapy completion rate
chemo_compleet	Neoadjuvant chemotherapy completion rate
CROSS_compleet	Neoadjuvant chemoradiotherapy completion rate
tumregres	Tumor regression grade primary tumor
pt	ypT
pn	ypN
yptn	ypTNM stage
chir_rad1	Surgical radicality
mort30	30-day post-operative mortality
mort90	90-day post-operative mortality
reden_geen_resectie	Reasons for not undergoing a surgical resection of primary tumor after nCRT
wk_eind_crt_resectie	Time between nCRT and resection (in weeks)
chir	Surgery (yes/no)

** The supplementary missing data assessment file contains the output of the assessment of missingness, this table is meant to show the meaning of each variable in that specific file.*

Supplementary Table S3. Reasons for not undergoing a surgical resection of primary tumor after neoadjuvant chemoradiotherapy (nCRT) (n=992)

	N	%
Active surveillance	368	37.1%
Disease progression	407	41.0%
Patient condition	90	9.1%
Patient request	88	8.9%
Other	39	3.9%
