# Supplementary material for: Real-world outcomes of the CROSS regimen in patients with resectable esophageal or gastro-esophageal junction adenocarcinoma: a nationwide cohort study in the Netherlands
Source: eClinicalMedicine. 2025 Jan 22;80:103067. doi: 10.1016/j.eclinm.2024.103067 (PMC11795631; doi:10.1016/j.eclinm.2024.103067)
Supplement: Supplementary Data [file mmc2.pdf]

Missing data matrix

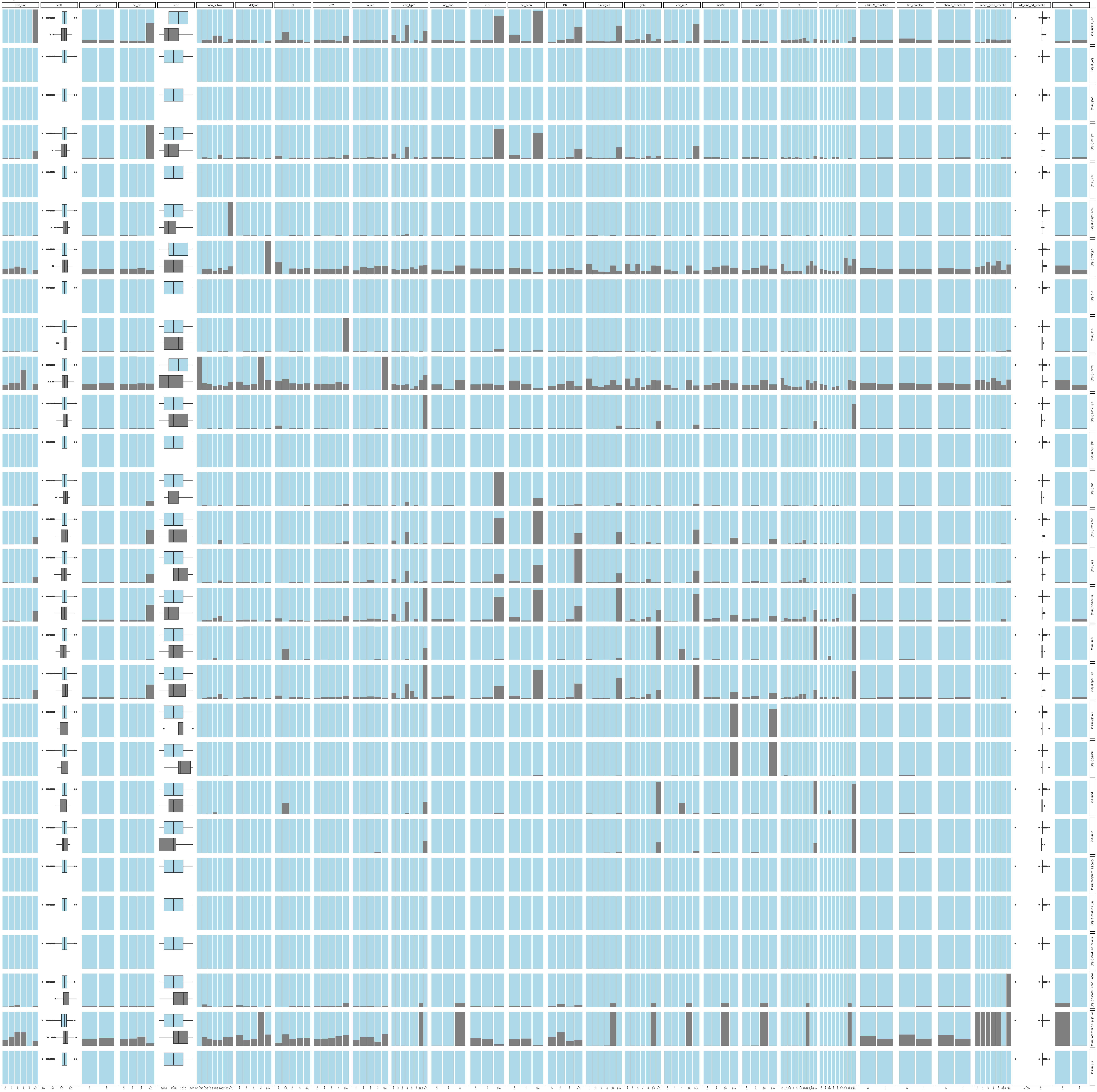

Missing data analysis: cci_cat		Not missing	Missing	p
leeft	Mean (SD)	66.0 (8.7)	65.0 (8.3)	0.161
gesl	1	3864 (96.5)	139 (3.5)	1.000
	2	736 (96.6)	26 (3.4)	
perf_stat	0	2470 (98.6)	36 (1.4)	0.934
	1	1666 (98.2)	30 (1.8)	
	2	135 (98.5)	2 (1.5)	
	3	5 (100.0)	0 (0.0)	
	4	1 (100.0)	0 (0.0)	
incjr	Mean (SD)	2018.5 (2.3)	2017.6 (2.1)	<0.001
topo_sublok	C153	1 (100.0)	0 (0.0)	<0.001
	C154	124 (96.1)	5 (3.9)	
	C155	3915 (96.9)	126 (3.1)	
	C158	18 (100.0)	0 (0.0)	
	C160	213 (87.7)	30 (12.3)	
	C167	279 (98.9)	3 (1.1)	
diffgrad	1	156 (96.3)	6 (3.7)	0.898
	2	1943 (96.1)	78 (3.9)	
	3	1730 (96.6)	61 (3.4)	
	4	1 (100.0)	0 (0.0)	
ct	1	10 (90.9)	1 (9.1)	0.785
	1B	3 (100.0)	0 (0.0)	
	2	1283 (96.7)	44 (3.3)	
	3	3250 (96.5)	119 (3.5)	
	4A	54 (98.2)	1 (1.8)	
cn2	0	1869 (96.6)	66 (3.4)	0.923
	1	1703 (96.7)	59 (3.3)	
	2	878 (96.4)	33 (3.6)	
	3	119 (97.5)	3 (2.5)	
lauren	1	2789 (96.5)	101 (3.5)	0.921
	2	643 (97.0)	20 (3.0)	
	3	121 (96.0)	5 (4.0)	
	4	200 (96.6)	7 (3.4)	
chir_type1	1	447 (84.8)	80 (15.2)	<0.001
	2	2298 (98.8)	29 (1.2)	
	3	715 (97.9)	15 (2.1)	
	4	51 (65.4)	27 (34.6)	
	5	44 (100.0)	0 (0.0)	
	7	43 (95.6)	2 (4.4)	
	888	981 (98.9)	11 (1.1)	
adj_nivo	0	3291 (96.0)	137 (4.0)	<0.001
	1	328 (95.1)	17 (4.9)	
	8	981 (98.9)	11 (1.1)	
eus	0	2009 (97.9)	43 (2.1)	0.002
	1	2588 (96.4)	98 (3.6)	
pet_scan	0	197 (89.1)	24 (10.9)	<0.001
	1	4381 (98.4)	69 (1.6)	
DR	0	2589 (99.2)	20 (0.8)	<0.001
	1	147 (97.4)	4 (2.6)	
	8	1758 (94.8)	97 (5.2)	
tumregres	1	701 (96.4)	26 (3.6)	0.001
	2	760 (97.9)	16 (2.1)	
	3	1786 (98.7)	24 (1.3)	
	4	210 (97.7)	5 (2.3)	
	88	981 (98.9)	11 (1.1)	
yptn	1	609 (95.5)	29 (4.5)	<0.001
	2	1457 (95.7)	66 (4.3)	
	3	86 (98.9)	1 (1.1)	

	4	1385 (96.4)	51 (3.6)	
	5	50 (92.6)	4 (7.4)	
	88	981 (98.9)	11 (1.1)	
chir_rad1	0	3181 (97.5)	80 (2.5)	0.063
	1	321 (98.5)	5 (1.5)	
	2	3 (100.0)	0 (0.0)	
	88	981 (98.9)	11 (1.1)	
mort30	0	3541 (95.9)	151 (4.1)	<0.001
	1	73 (96.1)	3 (3.9)	
	88	981 (98.9)	11 (1.1)	
mort90	0	3443 (95.9)	147 (4.1)	<0.001
	1	170 (96.0)	7 (4.0)	
	88	981 (98.9)	11 (1.1)	
pt	0	697 (95.9)	30 (4.1)	0.002
	1A	129 (97.0)	4 (3.0)	
	1B	527 (95.8)	23 (4.2)	
	2	705 (96.8)	23 (3.2)	
	3	1481 (95.5)	70 (4.5)	
	4A	37 (97.4)	1 (2.6)	
	4B	7 (100.0)	0 (0.0)	
	888	981 (98.9)	11 (1.1)	
	yis	5 (100.0)	0 (0.0)	
pn	0	2089 (95.6)	97 (4.4)	0.001
	1	842 (96.9)	27 (3.1)	
	1M	9 (100.0)	0 (0.0)	
	2	450 (95.9)	19 (4.1)	
	3	212 (95.1)	11 (4.9)	
	3A	4 (100.0)	0 (0.0)	
	3B	2 (100.0)	0 (0.0)	
	888	981 (98.9)	11 (1.1)	
CROSS_compleet	0	580 (97.5)	15 (2.5)	0.221
	1	4020 (96.4)	150 (3.6)	
RT_compleet	0	95 (97.9)	2 (2.1)	0.630
	1	4505 (96.5)	163 (3.5)	
chemo_compleet	0	518 (97.6)	13 (2.4)	0.218
	1	4082 (96.4)	152 (3.6)	
reden_geen_resectie	1	313 (100.0)	0 (0.0)	<0.001
	2	349 (98.9)	4 (1.1)	
	3	83 (97.6)	2 (2.4)	
	4	87 (100.0)	0 (0.0)	
	5	39 (100.0)	0 (0.0)	
	888	3619 (95.9)	154 (4.1)	
wk_eind_crt_resectie	Mean (SD)	11.0 (5.4)	9.6 (3.3)	0.002
chir	0	981 (98.9)	11 (1.1)	<0.001
	1	3619 (95.9)	154 (4.1)	

Missing data analysis: chir_rad1		Not missing	Missing	p
leeft	Mean (SD)	65.9 (8.7)	66.6 (8.5)	0.253
gesl	1	3859 (96.4)	144 (3.6)	0.058
	2	723 (94.9)	39 (5.1)	
cci_cat	0	2361 (97.6)	57 (2.4)	0.604
	1	1419 (97.2)	41 (2.8)	
	2	706 (97.8)	16 (2.2)	
incjr	Mean (SD)	2018.4 (2.3)	2018.6 (2.2)	0.367
perf_stat	0	2466 (98.4)	40 (1.6)	0.559
	1	1659 (97.8)	37 (2.2)	
	2	136 (99.3)	1 (0.7)	
	3	5 (100.0)	0 (0.0)	
	4	1 (100.0)	0 (0.0)	
topo_sublok	C153	1 (100.0)	0 (0.0)	<0.001
	C154	127 (98.4)	2 (1.6)	
	C155	3901 (96.5)	140 (3.5)	
	C158	17 (94.4)	1 (5.6)	
	C160	207 (85.2)	36 (14.8)	
	C167	278 (98.6)	4 (1.4)	
diffgrad	1	159 (98.1)	3 (1.9)	0.535
	2	1937 (95.8)	84 (4.2)	
	3	1716 (95.8)	75 (4.2)	
	4	1 (100.0)	0 (0.0)	
ct	1	10 (90.9)	1 (9.1)	0.811
	1B	3 (100.0)	0 (0.0)	
	2	1277 (96.2)	50 (3.8)	
	3	3238 (96.1)	131 (3.9)	
	4A	54 (98.2)	1 (1.8)	
cn2	0	1876 (97.0)	59 (3.0)	0.153
	1	1687 (95.7)	75 (4.3)	
	2	871 (95.6)	40 (4.4)	
	3	116 (95.1)	6 (4.9)	
chir_type1	1	436 (82.7)	91 (17.3)	<0.001
	2	2313 (99.4)	14 (0.6)	
	3	720 (98.6)	10 (1.4)	
	4	44 (56.4)	34 (43.6)	
	5	34 (77.3)	10 (22.7)	
	7	43 (95.6)	2 (4.4)	
	888	992 (100.0)	0 (0.0)	
lauren	1	2778 (96.1)	112 (3.9)	0.511
	2	635 (95.8)	28 (4.2)	
	3	118 (93.7)	8 (6.3)	
	4	197 (95.2)	10 (4.8)	
adj_nivo	0	3276 (95.6)	152 (4.4)	<0.001
	1	314 (91.0)	31 (9.0)	
	8	992 (100.0)	0 (0.0)	
eus	0	2009 (97.9)	43 (2.1)	<0.001
	1	2556 (95.2)	130 (4.8)	
pet_scan	0	202 (91.4)	19 (8.6)	<0.001
	1	4367 (98.1)	83 (1.9)	
DR	0	2567 (98.4)	42 (1.6)	<0.001
	1	149 (98.7)	2 (1.3)	
	8	1783 (96.1)	72 (3.9)	
tumregres	1	727 (100.0)	0 (0.0)	<0.001
	2	770 (99.2)	6 (0.8)	
	3	1786 (98.7)	24 (1.3)	
	4	212 (98.6)	3 (1.4)	
	88	992 (100.0)	0 (0.0)	

yptn	1	622 (97.5)	16 (2.5)	<0.001
	2	1451 (95.3)	72 (4.7)	
	3	85 (97.7)	2 (2.3)	
	4	1359 (94.6)	77 (5.4)	
	5	47 (87.0)	7 (13.0)	
	88	992 (100.0)	0 (0.0)	
mort30	0	3514 (95.2)	178 (4.8)	<0.001
	1	72 (94.7)	4 (5.3)	
	88	992 (100.0)	0 (0.0)	
mort90	0	3420 (95.3)	170 (4.7)	<0.001
	1	165 (93.2)	12 (6.8)	
	88	992 (100.0)	0 (0.0)	
pt	0	709 (97.5)	18 (2.5)	<0.001
	1A	126 (94.7)	7 (5.3)	
	1B	530 (96.4)	20 (3.6)	
	2	704 (96.7)	24 (3.3)	
	3	1452 (93.6)	99 (6.4)	
	4A	33 (86.8)	5 (13.2)	
	4B	6 (85.7)	1 (14.3)	
	888	992 (100.0)	0 (0.0)	
	yis	5 (100.0)	0 (0.0)	
pn	0	2097 (95.9)	89 (4.1)	<0.001
	1	823 (94.7)	46 (5.3)	
	1M	9 (100.0)	0 (0.0)	
	2	443 (94.5)	26 (5.5)	
	3	210 (94.2)	13 (5.8)	
	3A	4 (100.0)	0 (0.0)	
	3B	2 (100.0)	0 (0.0)	
	888	992 (100.0)	0 (0.0)	
CROSS_compleet	0	580 (97.5)	15 (2.5)	0.094
	1	4002 (96.0)	168 (4.0)	
RT_compleet	0	93 (95.9)	4 (4.1)	1.000
	1	4489 (96.2)	179 (3.8)	
chemo_compleet	0	520 (97.9)	11 (2.1)	0.033
	1	4062 (95.9)	172 (4.1)	
reden_geen_resectie	1	313 (100.0)	0 (0.0)	<0.001
	2	353 (100.0)	0 (0.0)	
	3	85 (100.0)	0 (0.0)	
	4	87 (100.0)	0 (0.0)	
	5	39 (100.0)	0 (0.0)	
	888	3590 (95.1)	183 (4.9)	
wk_eind_crt_resectie	Mean (SD)	11.0 (5.3)	10.0 (4.0)	0.013
chir	0	992 (100.0)	0 (0.0)	<0.001
	1	3590 (95.1)	183 (4.9)	

Missing data analysis: chir_type1		Not missing	Missing	p
leeft	Mean (SD)	65.9 (8.7)	68.4 (7.7)	0.177
gesl	1	3986 (99.6)	17 (0.4)	0.567
	2	757 (99.3)	5 (0.7)	
cci_cat	0	2410 (99.7)	8 (0.3)	0.404
	1	1451 (99.4)	9 (0.6)	
	2	718 (99.4)	4 (0.6)	
incjr	Mean (SD)	2018.4 (2.3)	2018.7 (2.4)	0.612
perf_stat	0	2499 (99.7)	7 (0.3)	0.921
	1	1690 (99.6)	6 (0.4)	
	2	136 (99.3)	1 (0.7)	
	3	5 (100.0)	0 (0.0)	
	4	1 (100.0)	0 (0.0)	
topo_sublok	C153	1 (100.0)	0 (0.0)	0.738
	C154	129 (100.0)	0 (0.0)	
	C155	4021 (99.5)	20 (0.5)	
	C158	18 (100.0)	0 (0.0)	
	C160	241 (99.2)	2 (0.8)	
	C167	282 (100.0)	0 (0.0)	
diffgrad	1	161 (99.4)	1 (0.6)	0.978
	2	2013 (99.6)	8 (0.4)	
	3	1784 (99.6)	7 (0.4)	
	4	1 (100.0)	0 (0.0)	
ct	1	10 (90.9)	1 (9.1)	0.001
	1B	3 (100.0)	0 (0.0)	
	2	1321 (99.5)	6 (0.5)	
	3	3354 (99.6)	15 (0.4)	
	4A	55 (100.0)	0 (0.0)	
cn2	0	1926 (99.5)	9 (0.5)	0.721
	1	1752 (99.4)	10 (0.6)	
	2	908 (99.7)	3 (0.3)	
	3	122 (100.0)	0 (0.0)	
lauren	1	2883 (99.8)	7 (0.2)	0.023
	2	661 (99.7)	2 (0.3)	
	3	126 (100.0)	0 (0.0)	
	4	204 (98.6)	3 (1.4)	
adj_nivo	0	3408 (99.4)	20 (0.6)	0.055
	1	343 (99.4)	2 (0.6)	
	8	992 (100.0)	0 (0.0)	
eus	0	2037 (99.3)	15 (0.7)	0.032
	1	2679 (99.7)	7 (0.3)	
pet_scan	0	218 (98.6)	3 (1.4)	0.121
	1	4432 (99.6)	18 (0.4)	
DR	0	2597 (99.5)	12 (0.5)	0.920
	1	150 (99.3)	1 (0.7)	
	8	1847 (99.6)	8 (0.4)	
yptn	1	634 (99.4)	4 (0.6)	0.131
	2	1517 (99.6)	6 (0.4)	
	3	86 (98.9)	1 (1.1)	
	4	1433 (99.8)	3 (0.2)	
	5	54 (100.0)	0 (0.0)	
	88	992 (100.0)	0 (0.0)	
mort30	0	3671 (99.4)	21 (0.6)	0.035
	1	75 (98.7)	1 (1.3)	
	88	992 (100.0)	0 (0.0)	
mort90	0	3571 (99.5)	19 (0.5)	0.005
	1	174 (98.3)	3 (1.7)	
	88	992 (100.0)	0 (0.0)	

pt	0	722 (99.3)	5 (0.7)	0.423
	1A	132 (99.2)	1 (0.8)	
	1B	549 (99.8)	1 (0.2)	
	2	726 (99.7)	2 (0.3)	
	3	1546 (99.7)	5 (0.3)	
	4A	38 (100.0)	0 (0.0)	
	4B	7 (100.0)	0 (0.0)	
	888	992 (100.0)	0 (0.0)	
	yis	5 (100.0)	0 (0.0)	
pn	0	2176 (99.5)	10 (0.5)	0.350
	1	865 (99.5)	4 (0.5)	
	1M	9 (100.0)	0 (0.0)	
	2	469 (100.0)	0 (0.0)	
	3	223 (100.0)	0 (0.0)	
	3A	4 (100.0)	0 (0.0)	
	3B	2 (100.0)	0 (0.0)	
	888	992 (100.0)	0 (0.0)	
CROSS_compleet	0	591 (99.3)	4 (0.7)	0.626
	1	4152 (99.6)	18 (0.4)	
RT_compleet	0	95 (97.9)	2 (2.1)	0.111
	1	4648 (99.6)	20 (0.4)	
chemo_compleet	0	529 (99.6)	2 (0.4)	1.000
	1	4214 (99.5)	20 (0.5)	
reden_geen_resectie	1	313 (100.0)	0 (0.0)	0.399
	2	353 (100.0)	0 (0.0)	
	3	85 (100.0)	0 (0.0)	
	4	87 (100.0)	0 (0.0)	
	5	39 (100.0)	0 (0.0)	
	888	3751 (99.4)	22 (0.6)	
wk_eind_crt_resectie	Mean (SD)	11.0 (5.3)	7.5 (4.7)	0.002
chir	0	992 (100.0)	0 (0.0)	0.032
	1	3751 (99.4)	22 (0.6)	

Missing data analysis: cn2		Not missing	Missing	p
leeft	Mean (SD)	65.9 (8.7)	67.7 (6.7)	0.226
gesl	1	3975 (99.3)	28 (0.7)	0.676
	2	755 (99.1)	7 (0.9)	
cci_cat	0	2404 (99.4)	14 (0.6)	0.694
	1	1449 (99.2)	11 (0.8)	
	2	716 (99.2)	6 (0.8)	
incjr	Mean (SD)	2018.4 (2.3)	2018.0 (2.4)	0.290
perf_stat	0	2493 (99.5)	13 (0.5)	0.819
	1	1682 (99.2)	14 (0.8)	
	2	136 (99.3)	1 (0.7)	
	3	5 (100.0)	0 (0.0)	
	4	1 (100.0)	0 (0.0)	
topo_sublok	C153	1 (100.0)	0 (0.0)	0.497
	C154	129 (100.0)	0 (0.0)	
	C155	4011 (99.3)	30 (0.7)	
	C158	18 (100.0)	0 (0.0)	
	C160	239 (98.4)	4 (1.6)	
	C167	281 (99.6)	1 (0.4)	
diffgrad	1	162 (100.0)	0 (0.0)	0.771
	2	2007 (99.3)	14 (0.7)	
	3	1779 (99.3)	12 (0.7)	
	4	1 (100.0)	0 (0.0)	
ct	1	11 (100.0)	0 (0.0)	0.885
	1B	3 (100.0)	0 (0.0)	
	2	1315 (99.1)	12 (0.9)	
	3	3346 (99.3)	23 (0.7)	
	4A	55 (100.0)	0 (0.0)	
lauren	1	2868 (99.2)	22 (0.8)	0.779
	2	658 (99.2)	5 (0.8)	
	3	126 (100.0)	0 (0.0)	
	4	205 (99.0)	2 (1.0)	
chir_type1	1	522 (99.1)	5 (0.9)	0.662
	2	2313 (99.4)	14 (0.6)	
	3	726 (99.5)	4 (0.5)	
	4	77 (98.7)	1 (1.3)	
	5	44 (100.0)	0 (0.0)	
	7	45 (100.0)	0 (0.0)	
	888	981 (98.9)	11 (1.1)	
adj_nivo	0	3405 (99.3)	23 (0.7)	0.220
	1	344 (99.7)	1 (0.3)	
	8	981 (98.9)	11 (1.1)	
eus	0	2034 (99.1)	18 (0.9)	0.258
	1	2671 (99.4)	15 (0.6)	
pet_scan	0	219 (99.1)	2 (0.9)	1.000
	1	4420 (99.3)	30 (0.7)	
DR	0	2593 (99.4)	16 (0.6)	0.497
	1	149 (98.7)	2 (1.3)	
	8	1840 (99.2)	15 (0.8)	
tumregres	1	721 (99.2)	6 (0.8)	0.157
	2	771 (99.4)	5 (0.6)	
	3	1804 (99.7)	6 (0.3)	
	4	214 (99.5)	1 (0.5)	
	88	981 (98.9)	11 (1.1)	
yptn	1	632 (99.1)	6 (0.9)	0.481
	2	1512 (99.3)	11 (0.7)	
	3	87 (100.0)	0 (0.0)	
	4	1429 (99.5)	7 (0.5)	

	5	54 (100.0)	0 (0.0)	
	88	981 (98.9)	11 (1.1)	
chir_rad1	0	3242 (99.4)	19 (0.6)	0.377
	1	324 (99.4)	2 (0.6)	
	2	3 (100.0)	0 (0.0)	
	88	981 (98.9)	11 (1.1)	
mort30	0	3668 (99.3)	24 (0.7)	0.243
	1	76 (100.0)	0 (0.0)	
	88	981 (98.9)	11 (1.1)	
mort90	0	3566 (99.3)	24 (0.7)	0.180
	1	177 (100.0)	0 (0.0)	
	88	981 (98.9)	11 (1.1)	
pt	0	721 (99.2)	6 (0.8)	0.877
	1A	133 (100.0)	0 (0.0)	
	1B	547 (99.5)	3 (0.5)	
	2	723 (99.3)	5 (0.7)	
	3	1541 (99.4)	10 (0.6)	
	4A	38 (100.0)	0 (0.0)	
	4B	7 (100.0)	0 (0.0)	
	888	981 (98.9)	11 (1.1)	
	yis	5 (100.0)	0 (0.0)	
pn	0	2169 (99.2)	17 (0.8)	0.759
	1	866 (99.7)	3 (0.3)	
	1M	9 (100.0)	0 (0.0)	
	2	466 (99.4)	3 (0.6)	
	3	222 (99.6)	1 (0.4)	
	3A	4 (100.0)	0 (0.0)	
	3B	2 (100.0)	0 (0.0)	
	888	981 (98.9)	11 (1.1)	
CROSS_compleet	0	588 (98.8)	7 (1.2)	0.274
	1	4142 (99.3)	28 (0.7)	
RT_compleet	0	95 (97.9)	2 (2.1)	0.344
	1	4635 (99.3)	33 (0.7)	
chemo_compleet	0	525 (98.9)	6 (1.1)	0.388
	1	4205 (99.3)	29 (0.7)	
reden_geen_resectie	1	310 (99.0)	3 (1.0)	0.563
	2	350 (99.2)	3 (0.8)	
	3	85 (100.0)	0 (0.0)	
	4	87 (100.0)	0 (0.0)	
	5	38 (97.4)	1 (2.6)	
	888	3749 (99.4)	24 (0.6)	
wk_eind_crt_resectie	Mean (SD)	10.9 (5.3)	10.0 (4.1)	0.403
chir	0	981 (98.9)	11 (1.1)	0.179
	1	3749 (99.4)	24 (0.6)	

Missing data analysis: diffgrad		Not missing	Missing	p
leeft	Mean (SD)	65.9 (8.6)	66.3 (8.7)	0.215
gesl	1	3334 (83.3)	669 (16.7)	0.607
	2	641 (84.1)	121 (15.9)	
cci_cat	0	2017 (83.4)	401 (16.6)	0.672
	1	1220 (83.6)	240 (16.4)	
	2	593 (82.1)	129 (17.9)	
incjr	Mean (SD)	2018.5 (2.3)	2018.2 (2.3)	0.001
perf_stat	0	2103 (83.9)	403 (16.1)	0.209
	1	1400 (82.5)	296 (17.5)	
	2	105 (76.6)	32 (23.4)	
	3	4 (80.0)	1 (20.0)	
	4	1 (100.0)	0 (0.0)	
topo_sublok	C153	1 (100.0)	0 (0.0)	0.732
	C154	108 (83.7)	21 (16.3)	
	C155	3370 (83.4)	671 (16.6)	
	C158	16 (88.9)	2 (11.1)	
	C160	198 (81.5)	45 (18.5)	
	C167	243 (86.2)	39 (13.8)	
ct	1	7 (63.6)	4 (36.4)	0.223
	1B	3 (100.0)	0 (0.0)	
	2	1092 (82.3)	235 (17.7)	
	3	2828 (83.9)	541 (16.1)	
	4A	45 (81.8)	10 (18.2)	
cn2	0	1603 (82.8)	332 (17.2)	0.722
	1	1474 (83.7)	288 (16.3)	
	2	770 (84.5)	141 (15.5)	
	3	102 (83.6)	20 (16.4)	
lauren	1	2555 (88.4)	335 (11.6)	<0.001
	2	515 (77.7)	148 (22.3)	
	3	103 (81.7)	23 (18.3)	
	4	153 (73.9)	54 (26.1)	
chir_type1	1	448 (85.0)	79 (15.0)	<0.001
	2	2018 (86.7)	309 (13.3)	
	3	623 (85.3)	107 (14.7)	
	4	66 (84.6)	12 (15.4)	
	5	35 (79.5)	9 (20.5)	
	7	38 (84.4)	7 (15.6)	
	888	731 (73.7)	261 (26.3)	
adj_nivo	0	2937 (85.7)	491 (14.3)	<0.001
	1	307 (89.0)	38 (11.0)	
	8	731 (73.7)	261 (26.3)	
eus	0	1689 (82.3)	363 (17.7)	0.082
	1	2263 (84.3)	423 (15.7)	
pet_scan	0	176 (79.6)	45 (20.4)	0.172
	1	3711 (83.4)	739 (16.6)	
DR	0	2212 (84.8)	397 (15.2)	0.009
	1	125 (82.8)	26 (17.2)	
	8	1508 (81.3)	347 (18.7)	
tumregres	1	500 (68.8)	227 (31.2)	<0.001
	2	666 (85.8)	110 (14.2)	
	3	1658 (91.6)	152 (8.4)	
	4	200 (93.0)	15 (7.0)	
	88	731 (73.7)	261 (26.3)	
yptn	1	438 (68.7)	200 (31.3)	<0.001
	2	1376 (90.3)	147 (9.7)	
	3	60 (69.0)	27 (31.0)	
	4	1295 (90.2)	141 (9.8)	

	5	49 (90.7)	5 (9.3)	
	88	731 (73.7)	261 (26.3)	
chir_rad1	0	2784 (85.4)	477 (14.6)	<0.001
	1	295 (90.5)	31 (9.5)	
	2	3 (100.0)	0 (0.0)	
	88	731 (73.7)	261 (26.3)	
mort30	0	3181 (86.2)	511 (13.8)	<0.001
	1	59 (77.6)	17 (22.4)	
	88	731 (73.7)	261 (26.3)	
mort90	0	3095 (86.2)	495 (13.8)	<0.001
	1	144 (81.4)	33 (18.6)	
	88	731 (73.7)	261 (26.3)	
pt	0	500 (68.8)	227 (31.2)	<0.001
	1A	119 (89.5)	14 (10.5)	
	1B	496 (90.2)	54 (9.8)	
	2	659 (90.5)	69 (9.5)	
	3	1401 (90.3)	150 (9.7)	
	4A	34 (89.5)	4 (10.5)	
	4B	7 (100.0)	0 (0.0)	
	888	731 (73.7)	261 (26.3)	
	yis	3 (60.0)	2 (40.0)	
pn	0	1836 (84.0)	350 (16.0)	<0.001
	1	764 (87.9)	105 (12.1)	
	1M	8 (88.9)	1 (11.1)	
	2	425 (90.6)	44 (9.4)	
	3	200 (89.7)	23 (10.3)	
	3A	4 (100.0)	0 (0.0)	
	3B	1 (50.0)	1 (50.0)	
	888	731 (73.7)	261 (26.3)	
CROSS_compleet	0	485 (81.5)	110 (18.5)	0.201
	1	3490 (83.7)	680 (16.3)	
RT_compleet	0	81 (83.5)	16 (16.5)	1.000
	1	3894 (83.4)	774 (16.6)	
chemo_compleet	0	429 (80.8)	102 (19.2)	0.096
	1	3546 (83.8)	688 (16.2)	
reden_geen_resectie	1	241 (77.0)	72 (23.0)	<0.001
	2	268 (75.9)	85 (24.1)	
	3	54 (63.5)	31 (36.5)	
	4	64 (73.6)	23 (26.4)	
	5	23 (59.0)	16 (41.0)	
	888	3244 (86.0)	529 (14.0)	
wk_eind_crt_resectie	Mean (SD)	11.0 (5.5)	10.5 (4.1)	0.055
chir	0	731 (73.7)	261 (26.3)	<0.001
	1	3244 (86.0)	529 (14.0)	

Missing data analysis: DR		Not missing	Missing	p
leeft	Mean (SD)	65.9 (8.7)	65.7 (8.3)	0.713
gesl	1	3875 (96.8)	128 (3.2)	0.736
	2	740 (97.1)	22 (2.9)	
cci_cat	0	2364 (97.8)	54 (2.2)	0.917
	1	1426 (97.7)	34 (2.3)	
	2	704 (97.5)	18 (2.5)	
incjr	Mean (SD)	2018.4 (2.3)	2019.5 (1.5)	<0.001
perf_stat	0	2449 (97.7)	57 (2.3)	0.047
	1	1676 (98.8)	20 (1.2)	
	2	137 (100.0)	0 (0.0)	
	3	5 (100.0)	0 (0.0)	
	4	1 (100.0)	0 (0.0)	
topo_sublok	C153	1 (100.0)	0 (0.0)	0.021
	C154	126 (97.7)	3 (2.3)	
	C155	3919 (97.0)	122 (3.0)	
	C158	18 (100.0)	0 (0.0)	
	C160	226 (93.0)	17 (7.0)	
	C167	275 (97.5)	7 (2.5)	
diffgrad	1	159 (98.1)	3 (1.9)	0.718
	2	1951 (96.5)	70 (3.5)	
	3	1734 (96.8)	57 (3.2)	
	4	1 (100.0)	0 (0.0)	
ct	1	11 (100.0)	0 (0.0)	0.673
	1B	3 (100.0)	0 (0.0)	
	2	1286 (96.9)	41 (3.1)	
	3	3260 (96.8)	109 (3.2)	
	4A	55 (100.0)	0 (0.0)	
cn2	0	1879 (97.1)	56 (2.9)	0.685
	1	1708 (96.9)	54 (3.1)	
	2	878 (96.4)	33 (3.6)	
	3	117 (95.9)	5 (4.1)	
chir_type1	1	470 (89.2)	57 (10.8)	<0.001
	2	2307 (99.1)	20 (0.9)	
	3	713 (97.7)	17 (2.3)	
	4	50 (64.1)	28 (35.9)	
	5	44 (100.0)	0 (0.0)	
	7	43 (95.6)	2 (4.4)	
	888	967 (97.5)	25 (2.5)	
lauren	1	2787 (96.4)	103 (3.6)	0.003
	2	647 (97.6)	16 (2.4)	
	3	116 (92.1)	10 (7.9)	
	4	205 (99.0)	2 (1.0)	
adj_nivo	0	3322 (96.9)	106 (3.1)	0.022
	1	326 (94.5)	19 (5.5)	
	8	967 (97.5)	25 (2.5)	
pet_scan	0	206 (93.2)	15 (6.8)	<0.001
	1	4365 (98.1)	85 (1.9)	
eus	0	2020 (98.4)	32 (1.6)	<0.001
	1	2575 (95.9)	111 (4.1)	
tumregres	1	713 (98.1)	14 (1.9)	0.267
	2	767 (98.8)	9 (1.2)	
	3	1781 (98.4)	29 (1.6)	
	4	211 (98.1)	4 (1.9)	
	88	967 (97.5)	25 (2.5)	
yptn	1	621 (97.3)	17 (2.7)	0.010
	2	1468 (96.4)	55 (3.6)	
	3	86 (98.9)	1 (1.1)	

	4	1391 (96.9)	45 (3.1)	
	5	48 (88.9)	6 (11.1)	
	88	967 (97.5)	25 (2.5)	
chir_rad1	0	3205 (98.3)	56 (1.7)	0.130
	1	324 (99.4)	2 (0.6)	
	2	3 (100.0)	0 (0.0)	
	88	967 (97.5)	25 (2.5)	
mort30	0	3570 (96.7)	122 (3.3)	0.420
	1	73 (96.1)	3 (3.9)	
	88	967 (97.5)	25 (2.5)	
mort90	0	3473 (96.7)	117 (3.3)	0.284
	1	169 (95.5)	8 (4.5)	
	88	967 (97.5)	25 (2.5)	
pt	0	709 (97.5)	18 (2.5)	0.283
	1A	128 (96.2)	5 (3.8)	
	1B	529 (96.2)	21 (3.8)	
	2	706 (97.0)	22 (3.0)	
	3	1497 (96.5)	54 (3.5)	
	4A	35 (92.1)	3 (7.9)	
	4B	6 (85.7)	1 (14.3)	
	888	967 (97.5)	25 (2.5)	
	yis	5 (100.0)	0 (0.0)	
pn	0	2112 (96.6)	74 (3.4)	0.909
	1	843 (97.0)	26 (3.0)	
	1M	9 (100.0)	0 (0.0)	
	2	452 (96.4)	17 (3.6)	
	3	215 (96.4)	8 (3.6)	
	3A	4 (100.0)	0 (0.0)	
	3B	2 (100.0)	0 (0.0)	
	888	967 (97.5)	25 (2.5)	
CROSS_compleet	0	585 (98.3)	10 (1.7)	0.039
	1	4030 (96.6)	140 (3.4)	
RT_compleet	0	96 (99.0)	1 (1.0)	0.361
	1	4519 (96.8)	149 (3.2)	
chemo_compleet	0	522 (98.3)	9 (1.7)	0.057
	1	4093 (96.7)	141 (3.3)	
reden_geen_resectie	1	303 (96.8)	10 (3.2)	0.130
	2	347 (98.3)	6 (1.7)	
	3	85 (100.0)	0 (0.0)	
	4	87 (100.0)	0 (0.0)	
	5	38 (97.4)	1 (2.6)	
	888	3648 (96.7)	125 (3.3)	
wk_eind_crt_resectie	Mean (SD)	10.9 (5.3)	11.0 (4.3)	0.830
chir	0	967 (97.5)	25 (2.5)	0.242
	1	3648 (96.7)	125 (3.3)	

Missing data analysis: eus		Not missing	Missing	p
leeft	Mean (SD)	65.9 (8.7)	67.6 (7.9)	0.315
gesl	1	3980 (99.4)	23 (0.6)	1.000
	2	758 (99.5)	4 (0.5)	
cci_cat	0	2416 (99.9)	2 (0.1)	0.746
	1	1459 (99.9)	1 (0.1)	
	2	722 (100.0)	0 (0.0)	
incjr	Mean (SD)	2018.4 (2.3)	2017.9 (1.9)	0.245
perf_stat	0	2503 (99.9)	3 (0.1)	0.997
	1	1694 (99.9)	2 (0.1)	
	2	137 (100.0)	0 (0.0)	
	3	5 (100.0)	0 (0.0)	
	4	1 (100.0)	0 (0.0)	
topo_sublok	C153	1 (100.0)	0 (0.0)	0.108
	C154	127 (98.4)	2 (1.6)	
	C155	4020 (99.5)	21 (0.5)	
	C158	18 (100.0)	0 (0.0)	
	C160	239 (98.4)	4 (1.6)	
	C167	282 (100.0)	0 (0.0)	
diffgrad	1	159 (98.1)	3 (1.9)	0.076
	2	2007 (99.3)	14 (0.7)	
	3	1785 (99.7)	6 (0.3)	
	4	1 (100.0)	0 (0.0)	
ct	1	11 (100.0)	0 (0.0)	0.743
	1B	3 (100.0)	0 (0.0)	
	2	1321 (99.5)	6 (0.5)	
	3	3349 (99.4)	20 (0.6)	
	4A	54 (98.2)	1 (1.8)	
cn2	0	1924 (99.4)	11 (0.6)	0.930
	1	1754 (99.5)	8 (0.5)	
	2	906 (99.5)	5 (0.5)	
	3	121 (99.2)	1 (0.8)	
chir_type1	1	511 (97.0)	16 (3.0)	<0.001
	2	2326 (100.0)	1 (0.0)	
	3	730 (100.0)	0 (0.0)	
	4	70 (89.7)	8 (10.3)	
	5	44 (100.0)	0 (0.0)	
	7	44 (97.8)	1 (2.2)	
	888	991 (99.9)	1 (0.1)	
lauren	1	2872 (99.4)	18 (0.6)	0.665
	2	661 (99.7)	2 (0.3)	
	3	125 (99.2)	1 (0.8)	
	4	205 (99.0)	2 (1.0)	
adj_nivo	0	3406 (99.4)	22 (0.6)	0.043
	1	341 (98.8)	4 (1.2)	
	8	991 (99.9)	1 (0.1)	
pet_scan	0	221 (100.0)	0 (0.0)	1.000
	1	4444 (99.9)	6 (0.1)	
DR	0	2607 (99.9)	2 (0.1)	<0.001
	1	149 (98.7)	2 (1.3)	
	8	1839 (99.1)	16 (0.9)	
tumregres	1	724 (99.6)	3 (0.4)	0.007
	2	776 (100.0)	0 (0.0)	
	3	1809 (99.9)	1 (0.1)	
	4	213 (99.1)	2 (0.9)	
	88	991 (99.9)	1 (0.1)	
yptn	1	635 (99.5)	3 (0.5)	0.178
	2	1511 (99.2)	12 (0.8)	

	3	87 (100.0)	0 (0.0)	
	4	1427 (99.4)	9 (0.6)	
	5	53 (98.1)	1 (1.9)	
	88	991 (99.9)	1 (0.1)	
chir_rad1	0	3246 (99.5)	15 (0.5)	0.439
	1	325 (99.7)	1 (0.3)	
	2	3 (100.0)	0 (0.0)	
	88	991 (99.9)	1 (0.1)	
mort30	0	3668 (99.3)	24 (0.7)	0.007
	1	74 (97.4)	2 (2.6)	
	88	991 (99.9)	1 (0.1)	
mort90	0	3566 (99.3)	24 (0.7)	0.065
	1	175 (98.9)	2 (1.1)	
	88	991 (99.9)	1 (0.1)	
pt	0	724 (99.6)	3 (0.4)	0.616
	1A	132 (99.2)	1 (0.8)	
	1B	546 (99.3)	4 (0.7)	
	2	723 (99.3)	5 (0.7)	
	3	1539 (99.2)	12 (0.8)	
	4A	38 (100.0)	0 (0.0)	
	4B	7 (100.0)	0 (0.0)	
	888	991 (99.9)	1 (0.1)	
	yis	5 (100.0)	0 (0.0)	
pn	0	2171 (99.3)	15 (0.7)	0.208
	1	866 (99.7)	3 (0.3)	
	1M	9 (100.0)	0 (0.0)	
	2	464 (98.9)	5 (1.1)	
	3	220 (98.7)	3 (1.3)	
	3A	4 (100.0)	0 (0.0)	
	3B	2 (100.0)	0 (0.0)	
	888	991 (99.9)	1 (0.1)	
CROSS_compleet	0	592 (99.5)	3 (0.5)	1.000
	1	4146 (99.4)	24 (0.6)	
RT_compleet	0	96 (99.0)	1 (1.0)	1.000
	1	4642 (99.4)	26 (0.6)	
chemo_compleet	0	529 (99.6)	2 (0.4)	0.755
	1	4209 (99.4)	25 (0.6)	
reden_geen_resectie	1	313 (100.0)	0 (0.0)	0.299
	2	353 (100.0)	0 (0.0)	
	3	85 (100.0)	0 (0.0)	
	4	87 (100.0)	0 (0.0)	
	5	39 (100.0)	0 (0.0)	
	888	3747 (99.3)	26 (0.7)	
wk_eind_crt_resectie	Mean (SD)	11.0 (5.3)	8.3 (3.0)	0.011
chir	0	991 (99.9)	1 (0.1)	0.050
	1	3747 (99.3)	26 (0.7)	

Missing data analysis: lauren		Not missing	Missing	p
leeft	Mean (SD)	65.8 (8.6)	66.3 (8.7)	0.201
gesl	1	3278 (81.9)	725 (18.1)	0.188
	2	608 (79.8)	154 (20.2)	
cci_cat	0	1980 (81.9)	438 (18.1)	0.573
	1	1194 (81.8)	266 (18.2)	
	2	579 (80.2)	143 (19.8)	
incjr	Mean (SD)	2018.6 (2.2)	2017.6 (2.4)	<0.001
perf_stat	0	2096 (83.6)	410 (16.4)	<0.001
	1	1341 (79.1)	355 (20.9)	
	2	107 (78.1)	30 (21.9)	
	3	2 (40.0)	3 (60.0)	
	4	1 (100.0)	0 (0.0)	
topo_sublok	C153	0 (0.0)	1 (100.0)	0.020
	C154	101 (78.3)	28 (21.7)	
	C155	3280 (81.2)	761 (18.8)	
	C158	16 (88.9)	2 (11.1)	
	C160	204 (84.0)	39 (16.0)	
	C167	246 (87.2)	36 (12.8)	
diffgrad	1	121 (74.7)	41 (25.3)	<0.001
	2	1735 (85.8)	286 (14.2)	
	3	1470 (82.1)	321 (17.9)	
	4	0 (0.0)	1 (100.0)	
ct	1	8 (72.7)	3 (27.3)	0.504
	1B	2 (66.7)	1 (33.3)	
	2	1065 (80.3)	262 (19.7)	
	3	2767 (82.1)	602 (17.9)	
	4A	44 (80.0)	11 (20.0)	
cn2	0	1599 (82.6)	336 (17.4)	0.217
	1	1430 (81.2)	332 (18.8)	
	2	735 (80.7)	176 (19.3)	
	3	93 (76.2)	29 (23.8)	
chir_type1	1	426 (80.8)	101 (19.2)	<0.001
	2	1981 (85.1)	346 (14.9)	
	3	622 (85.2)	108 (14.8)	
	4	65 (83.3)	13 (16.7)	
	5	42 (95.5)	2 (4.5)	
	7	40 (88.9)	5 (11.1)	
	888	698 (70.4)	294 (29.6)	
adj_nivo	0	2851 (83.2)	577 (16.8)	<0.001
	1	337 (97.7)	8 (2.3)	
	8	698 (70.4)	294 (29.6)	
eus	0	1704 (83.0)	348 (17.0)	0.021
	1	2159 (80.4)	527 (19.6)	
pet_scan	0	158 (71.5)	63 (28.5)	<0.001
	1	3639 (81.8)	811 (18.2)	
DR	0	2274 (87.2)	335 (12.8)	<0.001
	1	124 (82.1)	27 (17.9)	
	8	1357 (73.2)	498 (26.8)	
tumregres	1	475 (65.3)	252 (34.7)	<0.001
	2	685 (88.3)	91 (11.7)	
	3	1637 (90.4)	173 (9.6)	
	4	183 (85.1)	32 (14.9)	
	88	698 (70.4)	294 (29.6)	
yptn	1	418 (65.5)	220 (34.5)	<0.001
	2	1354 (88.9)	169 (11.1)	
	3	55 (63.2)	32 (36.8)	
	4	1290 (89.8)	146 (10.2)	

	5	46 (85.2)	8 (14.8)	
	88	698 (70.4)	294 (29.6)	
chir_rad1	0	2725 (83.6)	536 (16.4)	<0.001
	1	302 (92.6)	24 (7.4)	
	2	3 (100.0)	0 (0.0)	
	88	698 (70.4)	294 (29.6)	
mort30	0	3125 (84.6)	567 (15.4)	<0.001
	1	59 (77.6)	17 (22.4)	
	88	698 (70.4)	294 (29.6)	
mort90	0	3033 (84.5)	557 (15.5)	<0.001
	1	150 (84.7)	27 (15.3)	
	88	698 (70.4)	294 (29.6)	
pt	0	474 (65.2)	253 (34.8)	<0.001
	1A	113 (85.0)	20 (15.0)	
	1B	484 (88.0)	66 (12.0)	
	2	653 (89.7)	75 (10.3)	
	3	1394 (89.9)	157 (10.1)	
	4A	34 (89.5)	4 (10.5)	
	4B	7 (100.0)	0 (0.0)	
	888	698 (70.4)	294 (29.6)	
	yis	4 (80.0)	1 (20.0)	
pn	0	1791 (81.9)	395 (18.1)	<0.001
	1	748 (86.1)	121 (13.9)	
	1M	9 (100.0)	0 (0.0)	
	2	429 (91.5)	40 (8.5)	
	3	197 (88.3)	26 (11.7)	
	3A	4 (100.0)	0 (0.0)	
	3B	2 (100.0)	0 (0.0)	
	888	698 (70.4)	294 (29.6)	
CROSS_compleet	0	468 (78.7)	127 (21.3)	0.059
	1	3418 (82.0)	752 (18.0)	
RT_compleet	0	77 (79.4)	20 (20.6)	0.671
	1	3809 (81.6)	859 (18.4)	
chemo_compleet	0	418 (78.7)	113 (21.3)	0.084
	1	3468 (81.9)	766 (18.1)	
reden_geen_resectie	1	222 (70.9)	91 (29.1)	<0.001
	2	250 (70.8)	103 (29.2)	
	3	64 (75.3)	21 (24.7)	
	4	55 (63.2)	32 (36.8)	
	5	28 (71.8)	11 (28.2)	
	888	3188 (84.5)	585 (15.5)	
wk_eind_crt_resectie	Mean (SD)	11.1 (5.6)	10.1 (3.5)	<0.001
chir	0	698 (70.4)	294 (29.6)	<0.001
	1	3188 (84.5)	585 (15.5)	

Missing data analysis: mort30		Not missing	Missing	p
leeft	Mean (SD)	65.9 (8.7)	65.2 (10.6)	0.852
gesl	1	3999 (99.9)	4 (0.1)	1.000
	2	761 (99.9)	1 (0.1)	
cci_cat	0	2415 (99.9)	3 (0.1)	0.623
	1	1458 (99.9)	2 (0.1)	
	2	722 (100.0)	0 (0.0)	
incjr	Mean (SD)	2018.4 (2.3)	2019.2 (2.2)	0.454
perf_stat	0	2503 (99.9)	3 (0.1)	0.997
	1	1694 (99.9)	2 (0.1)	
	2	137 (100.0)	0 (0.0)	
	3	5 (100.0)	0 (0.0)	
	4	1 (100.0)	0 (0.0)	
topo_sublok	C153	1 (100.0)	0 (0.0)	0.838
	C154	129 (100.0)	0 (0.0)	
	C155	4037 (99.9)	4 (0.1)	
	C158	18 (100.0)	0 (0.0)	
	C160	243 (100.0)	0 (0.0)	
	C167	281 (99.6)	1 (0.4)	
diffgrad	1	162 (100.0)	0 (0.0)	0.806
	2	2018 (99.9)	3 (0.1)	
	3	1790 (99.9)	1 (0.1)	
	4	1 (100.0)	0 (0.0)	
ct	1	11 (100.0)	0 (0.0)	0.627
	1B	3 (100.0)	0 (0.0)	
	2	1324 (99.8)	3 (0.2)	
	3	3367 (99.9)	2 (0.1)	
	4A	55 (100.0)	0 (0.0)	
cn2	0	1933 (99.9)	2 (0.1)	0.986
	1	1760 (99.9)	2 (0.1)	
	2	910 (99.9)	1 (0.1)	
	3	122 (100.0)	0 (0.0)	
chir_type1	1	526 (99.8)	1 (0.2)	0.767
	2	2323 (99.8)	4 (0.2)	
	3	730 (100.0)	0 (0.0)	
	4	78 (100.0)	0 (0.0)	
	5	44 (100.0)	0 (0.0)	
	7	45 (100.0)	0 (0.0)	
	888	992 (100.0)	0 (0.0)	
lauren	1	2886 (99.9)	4 (0.1)	0.710
	2	663 (100.0)	0 (0.0)	
	3	126 (100.0)	0 (0.0)	
	4	207 (100.0)	0 (0.0)	
adj_nivo	0	3423 (99.9)	5 (0.1)	0.377
	1	345 (100.0)	0 (0.0)	
	8	992 (100.0)	0 (0.0)	
eus	0	2049 (99.9)	3 (0.1)	0.763
	1	2684 (99.9)	2 (0.1)	
pet_scan	0	221 (100.0)	0 (0.0)	1.000
	1	4446 (99.9)	4 (0.1)	
DR	0	2605 (99.8)	4 (0.2)	0.560
	1	151 (100.0)	0 (0.0)	
	8	1854 (99.9)	1 (0.1)	
tumregres	1	727 (100.0)	0 (0.0)	0.545
	2	775 (99.9)	1 (0.1)	
	3	1807 (99.8)	3 (0.2)	
	4	215 (100.0)	0 (0.0)	
	88	992 (100.0)	0 (0.0)	

yptn	1	637 (99.8)	1 (0.2)	0.694
	2	1522 (99.9)	1 (0.1)	
	3	87 (100.0)	0 (0.0)	
	4	1433 (99.8)	3 (0.2)	
	5	54 (100.0)	0 (0.0)	
	88	992 (100.0)	0 (0.0)	
chir_rad1	0	3259 (99.9)	2 (0.1)	0.009
	1	324 (99.4)	2 (0.6)	
	2	3 (100.0)	0 (0.0)	
	88	992 (100.0)	0 (0.0)	
pt	0	726 (99.9)	1 (0.1)	0.898
	1A	133 (100.0)	0 (0.0)	
	1B	549 (99.8)	1 (0.2)	
	2	728 (100.0)	0 (0.0)	
	3	1548 (99.8)	3 (0.2)	
	4A	38 (100.0)	0 (0.0)	
	4B	7 (100.0)	0 (0.0)	
	888	992 (100.0)	0 (0.0)	
	yis	5 (100.0)	0 (0.0)	
pn	0	2184 (99.9)	2 (0.1)	0.546
	1	868 (99.9)	1 (0.1)	
	1M	9 (100.0)	0 (0.0)	
	2	467 (99.6)	2 (0.4)	
	3	223 (100.0)	0 (0.0)	
	3A	4 (100.0)	0 (0.0)	
	3B	2 (100.0)	0 (0.0)	
	888	992 (100.0)	0 (0.0)	
CROSS_compleet	0	593 (99.7)	2 (0.3)	0.236
	1	4167 (99.9)	3 (0.1)	
RT_compleet	0	96 (99.0)	1 (1.0)	0.207
	1	4664 (99.9)	4 (0.1)	
chemo_compleet	0	530 (99.8)	1 (0.2)	1.000
	1	4230 (99.9)	4 (0.1)	
reden_geen_resectie	1	313 (100.0)	0 (0.0)	0.948
	2	353 (100.0)	0 (0.0)	
	3	85 (100.0)	0 (0.0)	
	4	87 (100.0)	0 (0.0)	
	5	39 (100.0)	0 (0.0)	
	888	3768 (99.9)	5 (0.1)	
wk_eind_crt_resectie	Mean (SD)	10.9 (5.2)	19.5 (22.5)	<0.001
chir	0	992 (100.0)	0 (0.0)	0.551
	1	3768 (99.9)	5 (0.1)	

Missing data analysis: mort90		Not missing	Missing	p
leeft	Mean (SD)	65.9 (8.7)	66.7 (10.1)	0.833
gesl	1	3998 (99.9)	5 (0.1)	1.000
	2	761 (99.9)	1 (0.1)	
cci_cat	0	2414 (99.8)	4 (0.2)	0.556
	1	1458 (99.9)	2 (0.1)	
	2	722 (100.0)	0 (0.0)	
incjr	Mean (SD)	2018.4 (2.3)	2019.7 (2.3)	0.186
perf_stat	0	2502 (99.8)	4 (0.2)	0.988
	1	1694 (99.9)	2 (0.1)	
	2	137 (100.0)	0 (0.0)	
	3	5 (100.0)	0 (0.0)	
	4	1 (100.0)	0 (0.0)	
topo_sublok	C153	1 (100.0)	0 (0.0)	0.895
	C154	129 (100.0)	0 (0.0)	
	C155	4036 (99.9)	5 (0.1)	
	C158	18 (100.0)	0 (0.0)	
	C160	243 (100.0)	0 (0.0)	
	C167	281 (99.6)	1 (0.4)	
diffgrad	1	162 (100.0)	0 (0.0)	0.628
	2	2017 (99.8)	4 (0.2)	
	3	1790 (99.9)	1 (0.1)	
	4	1 (100.0)	0 (0.0)	
ct	1	11 (100.0)	0 (0.0)	0.340
	1B	3 (100.0)	0 (0.0)	
	2	1323 (99.7)	4 (0.3)	
	3	3367 (99.9)	2 (0.1)	
	4A	55 (100.0)	0 (0.0)	
cn2	0	1932 (99.8)	3 (0.2)	0.956
	1	1760 (99.9)	2 (0.1)	
	2	910 (99.9)	1 (0.1)	
	3	122 (100.0)	0 (0.0)	
chir_type1	1	526 (99.8)	1 (0.2)	0.677
	2	2322 (99.8)	5 (0.2)	
	3	730 (100.0)	0 (0.0)	
	4	78 (100.0)	0 (0.0)	
	5	44 (100.0)	0 (0.0)	
	7	45 (100.0)	0 (0.0)	
	888	992 (100.0)	0 (0.0)	
lauren	1	2885 (99.8)	5 (0.2)	0.631
	2	663 (100.0)	0 (0.0)	
	3	126 (100.0)	0 (0.0)	
	4	207 (100.0)	0 (0.0)	
adj_nivo	0	3422 (99.8)	6 (0.2)	0.310
	1	345 (100.0)	0 (0.0)	
	8	992 (100.0)	0 (0.0)	
eus	0	2048 (99.8)	4 (0.2)	0.457
	1	2684 (99.9)	2 (0.1)	
pet_scan	0	221 (100.0)	0 (0.0)	1.000
	1	4445 (99.9)	5 (0.1)	
DR	0	2604 (99.8)	5 (0.2)	0.409
	1	151 (100.0)	0 (0.0)	
	8	1854 (99.9)	1 (0.1)	
tumregres	1	727 (100.0)	0 (0.0)	0.385
	2	775 (99.9)	1 (0.1)	
	3	1806 (99.8)	4 (0.2)	
	4	215 (100.0)	0 (0.0)	
	88	992 (100.0)	0 (0.0)	

yptn	1	637 (99.8)	1 (0.2)	0.814
	2	1521 (99.9)	2 (0.1)	
	3	87 (100.0)	0 (0.0)	
	4	1433 (99.8)	3 (0.2)	
	5	54 (100.0)	0 (0.0)	
	88	992 (100.0)	0 (0.0)	
chir_rad1	0	3258 (99.9)	3 (0.1)	0.032
	1	324 (99.4)	2 (0.6)	
	2	3 (100.0)	0 (0.0)	
	88	992 (100.0)	0 (0.0)	
mort30	0	3691 (100.0)	1 (0.0)	0.865
	1	76 (100.0)	0 (0.0)	
	88	992 (100.0)	0 (0.0)	
pt	0	726 (99.9)	1 (0.1)	0.533
	1A	132 (99.2)	1 (0.8)	
	1B	549 (99.8)	1 (0.2)	
	2	728 (100.0)	0 (0.0)	
	3	1548 (99.8)	3 (0.2)	
	4A	38 (100.0)	0 (0.0)	
	4B	7 (100.0)	0 (0.0)	
	888	992 (100.0)	0 (0.0)	
	yis	5 (100.0)	0 (0.0)	
pn	0	2183 (99.9)	3 (0.1)	0.668
	1	868 (99.9)	1 (0.1)	
	1M	9 (100.0)	0 (0.0)	
	2	467 (99.6)	2 (0.4)	
	3	223 (100.0)	0 (0.0)	
	3A	4 (100.0)	0 (0.0)	
	3B	2 (100.0)	0 (0.0)	
	888	992 (100.0)	0 (0.0)	
CROSS_compleet	0	593 (99.7)	2 (0.3)	0.354
	1	4166 (99.9)	4 (0.1)	
RT_compleet	0	96 (99.0)	1 (1.0)	0.274
	1	4663 (99.9)	5 (0.1)	
chemo_compleet	0	530 (99.8)	1 (0.2)	1.000
	1	4229 (99.9)	5 (0.1)	
reden_geen_resectie	1	313 (100.0)	0 (0.0)	0.925
	2	353 (100.0)	0 (0.0)	
	3	85 (100.0)	0 (0.0)	
	4	87 (100.0)	0 (0.0)	
	5	39 (100.0)	0 (0.0)	
	888	3767 (99.8)	6 (0.2)	
wk_eind_crt_resectie	Mean (SD)	10.9 (5.2)	18.1 (20.4)	0.001
chir	0	992 (100.0)	0 (0.0)	0.451
	1	3767 (99.8)	6 (0.2)	

Missing data analysis: perf_stat		Not missing	Missing	p
leeft	Mean (SD)	66.0 (8.7)	65.5 (8.3)	0.331
gesl	1	3659 (91.4)	344 (8.6)	0.245
	2	686 (90.0)	76 (10.0)	
cci_cat	0	2245 (92.8)	173 (7.2)	0.927
	1	1359 (93.1)	101 (6.9)	
	2	673 (93.2)	49 (6.8)	
incjr	Mean (SD)	2018.5 (2.3)	2017.7 (2.1)	<0.001
topo_sublok	C153	1 (100.0)	0 (0.0)	<0.001
	C154	116 (89.9)	13 (10.1)	
	C155	3704 (91.7)	337 (8.3)	
	C158	14 (77.8)	4 (22.2)	
	C160	192 (79.0)	51 (21.0)	
	C167	273 (96.8)	9 (3.2)	
diffgrad	1	147 (90.7)	15 (9.3)	0.709
	2	1827 (90.4)	194 (9.6)	
	3	1638 (91.5)	153 (8.5)	
	4	1 (100.0)	0 (0.0)	
ct	1	10 (90.9)	1 (9.1)	0.238
	1B	2 (66.7)	1 (33.3)	
	2	1199 (90.4)	128 (9.6)	
	3	3081 (91.5)	288 (8.5)	
	4A	53 (96.4)	2 (3.6)	
cn2	0	1757 (90.8)	178 (9.2)	0.336
	1	1622 (92.1)	140 (7.9)	
	2	824 (90.5)	87 (9.5)	
	3	114 (93.4)	8 (6.6)	
lauren	1	2633 (91.1)	257 (8.9)	0.849
	2	611 (92.2)	52 (7.8)	
	3	115 (91.3)	11 (8.7)	
	4	188 (90.8)	19 (9.2)	
chir_type1	1	398 (75.5)	129 (24.5)	<0.001
	2	2197 (94.4)	130 (5.6)	
	3	678 (92.9)	52 (7.1)	
	4	37 (47.4)	41 (52.6)	
	5	44 (100.0)	0 (0.0)	
	7	41 (91.1)	4 (8.9)	
	888	936 (94.4)	56 (5.6)	
adj_nivo	0	3093 (90.2)	335 (9.8)	<0.001
	1	316 (91.6)	29 (8.4)	
	8	936 (94.4)	56 (5.6)	
eus	0	1876 (91.4)	176 (8.6)	0.741
	1	2464 (91.7)	222 (8.3)	
pet_scan	0	168 (76.0)	53 (24.0)	<0.001
	1	4172 (93.8)	278 (6.2)	
DR	0	2515 (96.4)	94 (3.6)	<0.001
	1	138 (91.4)	13 (8.6)	
	8	1615 (87.1)	240 (12.9)	
tumregres	1	675 (92.8)	52 (7.2)	0.429
	2	719 (92.7)	57 (7.3)	
	3	1692 (93.5)	118 (6.5)	
	4	205 (95.3)	10 (4.7)	
	88	936 (94.4)	56 (5.6)	
yptn	1	588 (92.2)	50 (7.8)	<0.001
	2	1368 (89.8)	155 (10.2)	
	3	77 (88.5)	10 (11.5)	
	4	1305 (90.9)	131 (9.1)	
	5	40 (74.1)	14 (25.9)	

	88	936 (94.4)	56 (5.6)	
chir_rad1	0	3029 (92.9)	232 (7.1)	0.276
	1	299 (91.7)	27 (8.3)	
	2	3 (100.0)	0 (0.0)	
	88	936 (94.4)	56 (5.6)	
mort30	0	3335 (90.3)	357 (9.7)	<0.001
	1	69 (90.8)	7 (9.2)	
	88	936 (94.4)	56 (5.6)	
mort90	0	3244 (90.4)	346 (9.6)	<0.001
	1	159 (89.8)	18 (10.2)	
	88	936 (94.4)	56 (5.6)	
pt	0	667 (91.7)	60 (8.3)	0.009
	1A	123 (92.5)	10 (7.5)	
	1B	494 (89.8)	56 (10.2)	
	2	659 (90.5)	69 (9.5)	
	3	1392 (89.7)	159 (10.3)	
	4A	33 (86.8)	5 (13.2)	
	4B	6 (85.7)	1 (14.3)	
	888	936 (94.4)	56 (5.6)	
	yis	5 (100.0)	0 (0.0)	
pn	0	1979 (90.5)	207 (9.5)	0.014
	1	784 (90.2)	85 (9.8)	
	1M	9 (100.0)	0 (0.0)	
	2	422 (90.0)	47 (10.0)	
	3	200 (89.7)	23 (10.3)	
	3A	4 (100.0)	0 (0.0)	
	3B	2 (100.0)	0 (0.0)	
	888	936 (94.4)	56 (5.6)	
CROSS_compleet	0	540 (90.8)	55 (9.2)	0.751
	1	3805 (91.2)	365 (8.8)	
RT_compleet	0	84 (86.6)	13 (13.4)	0.153
	1	4261 (91.3)	407 (8.7)	
chemo_compleet	0	484 (91.1)	47 (8.9)	1.000
	1	3861 (91.2)	373 (8.8)	
reden_geen_resectie	1	304 (97.1)	9 (2.9)	<0.001
	2	339 (96.0)	14 (4.0)	
	3	76 (89.4)	9 (10.6)	
	4	78 (89.7)	9 (10.3)	
	5	36 (92.3)	3 (7.7)	
	888	3409 (90.4)	364 (9.6)	
wk_eind_crt_resectie	Mean (SD)	11.1 (5.4)	9.9 (4.0)	<0.001
chir	0	936 (94.4)	56 (5.6)	<0.001
	1	3409 (90.4)	364 (9.6)	

Missing data analysis: pet_scan		Not missing	Missing	p
leeft	Mean (SD)	65.9 (8.7)	66.0 (8.3)	0.940
gesl	1	3926 (98.1)	77 (1.9)	0.677
	2	745 (97.8)	17 (2.2)	
cci_cat	0	2405 (99.5)	13 (0.5)	0.354
	1	1452 (99.5)	8 (0.5)	
	2	721 (99.9)	1 (0.1)	
incjr	Mean (SD)	2018.4 (2.3)	2018.5 (2.1)	0.679
perf_stat	0	2503 (99.9)	3 (0.1)	0.997
	1	1694 (99.9)	2 (0.1)	
	2	137 (100.0)	0 (0.0)	
	3	5 (100.0)	0 (0.0)	
	4	1 (100.0)	0 (0.0)	
topo_sublok	C153	1 (100.0)	0 (0.0)	<0.001
	C154	126 (97.7)	3 (2.3)	
	C155	3980 (98.5)	61 (1.5)	
	C158	18 (100.0)	0 (0.0)	
	C160	213 (87.7)	30 (12.3)	
	C167	282 (100.0)	0 (0.0)	
diffgrad	1	161 (99.4)	1 (0.6)	0.491
	2	1972 (97.6)	49 (2.4)	
	3	1753 (97.9)	38 (2.1)	
	4	1 (100.0)	0 (0.0)	
ct	1	11 (100.0)	0 (0.0)	0.966
	1B	3 (100.0)	0 (0.0)	
	2	1303 (98.2)	24 (1.8)	
	3	3300 (98.0)	69 (2.0)	
	4A	54 (98.2)	1 (1.8)	
cn2	0	1903 (98.3)	32 (1.7)	0.718
	1	1725 (97.9)	37 (2.1)	
	2	892 (97.9)	19 (2.1)	
	3	119 (97.5)	3 (2.5)	
chir_type1	1	467 (88.6)	60 (11.4)	<0.001
	2	2326 (100.0)	1 (0.0)	
	3	730 (100.0)	0 (0.0)	
	4	49 (62.8)	29 (37.2)	
	5	44 (100.0)	0 (0.0)	
	7	43 (95.6)	2 (4.4)	
	888	991 (99.9)	1 (0.1)	
lauren	1	2823 (97.7)	67 (2.3)	0.219
	2	650 (98.0)	13 (2.0)	
	3	120 (95.2)	6 (4.8)	
	4	204 (98.6)	3 (1.4)	
adj_nivo	0	3353 (97.8)	75 (2.2)	<0.001
	1	327 (94.8)	18 (5.2)	
	8	991 (99.9)	1 (0.1)	
eus	0	2052 (100.0)	0 (0.0)	<0.001
	1	2613 (97.3)	73 (2.7)	
DR	0	2607 (99.9)	2 (0.1)	<0.001
	1	149 (98.7)	2 (1.3)	
	8	1815 (97.8)	40 (2.2)	
tumregres	1	724 (99.6)	3 (0.4)	0.091
	2	776 (100.0)	0 (0.0)	
	3	1809 (99.9)	1 (0.1)	
	4	214 (99.5)	1 (0.5)	
	88	991 (99.9)	1 (0.1)	
yptn	1	629 (98.6)	9 (1.4)	<0.001
	2	1479 (97.1)	44 (2.9)	

	3	86 (98.9)	1 (1.1)	
	4	1402 (97.6)	34 (2.4)	
	5	50 (92.6)	4 (7.4)	
	88	991 (99.9)	1 (0.1)	
chir_rad1	0	3249 (99.6)	12 (0.4)	0.403
	1	326 (100.0)	0 (0.0)	
	2	3 (100.0)	0 (0.0)	
	88	991 (99.9)	1 (0.1)	
mort30	0	3601 (97.5)	91 (2.5)	<0.001
	1	75 (98.7)	1 (1.3)	
	88	991 (99.9)	1 (0.1)	
mort90	0	3501 (97.5)	89 (2.5)	<0.001
	1	174 (98.3)	3 (1.7)	
	88	991 (99.9)	1 (0.1)	
pt	0	717 (98.6)	10 (1.4)	<0.001
	1A	131 (98.5)	2 (1.5)	
	1B	536 (97.5)	14 (2.5)	
	2	713 (97.9)	15 (2.1)	
	3	1503 (96.9)	48 (3.1)	
	4A	36 (94.7)	2 (5.3)	
	4B	6 (85.7)	1 (14.3)	
	888	991 (99.9)	1 (0.1)	
	yis	5 (100.0)	0 (0.0)	
pn	0	2133 (97.6)	53 (2.4)	0.001
	1	848 (97.6)	21 (2.4)	
	1M	9 (100.0)	0 (0.0)	
	2	458 (97.7)	11 (2.3)	
	3	215 (96.4)	8 (3.6)	
	3A	4 (100.0)	0 (0.0)	
	3B	2 (100.0)	0 (0.0)	
	888	991 (99.9)	1 (0.1)	
CROSS_compleet	0	587 (98.7)	8 (1.3)	0.308
	1	4084 (97.9)	86 (2.1)	
RT_compleet	0	95 (97.9)	2 (2.1)	1.000
	1	4576 (98.0)	92 (2.0)	
chemo_compleet	0	525 (98.9)	6 (1.1)	0.188
	1	4146 (97.9)	88 (2.1)	
reden_geen_resectie	1	313 (100.0)	0 (0.0)	0.001
	2	353 (100.0)	0 (0.0)	
	3	85 (100.0)	0 (0.0)	
	4	87 (100.0)	0 (0.0)	
	5	39 (100.0)	0 (0.0)	
	888	3680 (97.5)	93 (2.5)	
wk_eind_crt_resectie	Mean (SD)	11.0 (5.3)	10.1 (3.8)	0.111
chir	0	991 (99.9)	1 (0.1)	<0.001
	1	3680 (97.5)	93 (2.5)	

Missing data analysis: pn		Not missing	Missing	p
leeft	Mean (SD)	65.9 (8.7)	66.6 (9.0)	0.784
gesl	1	3994 (99.8)	9 (0.2)	1.000
	2	760 (99.7)	2 (0.3)	
cci_cat	0	2414 (99.8)	4 (0.2)	0.457
	1	1456 (99.7)	4 (0.3)	
	2	719 (99.6)	3 (0.4)	
incjr	Mean (SD)	2018.4 (2.3)	2017.5 (2.3)	0.154
perf_stat	0	2502 (99.8)	4 (0.2)	0.878
	1	1691 (99.7)	5 (0.3)	
	2	137 (100.0)	0 (0.0)	
	3	5 (100.0)	0 (0.0)	
	4	1 (100.0)	0 (0.0)	
topo_sublok	C153	1 (100.0)	0 (0.0)	0.871
	C154	129 (100.0)	0 (0.0)	
	C155	4030 (99.7)	11 (0.3)	
	C158	18 (100.0)	0 (0.0)	
	C160	243 (100.0)	0 (0.0)	
	C167	282 (100.0)	0 (0.0)	
diffgrad	1	162 (100.0)	0 (0.0)	0.867
	2	2017 (99.8)	4 (0.2)	
	3	1789 (99.9)	2 (0.1)	
	4	1 (100.0)	0 (0.0)	
ct	1	11 (100.0)	0 (0.0)	0.334
	1B	3 (100.0)	0 (0.0)	
	2	1327 (100.0)	0 (0.0)	
	3	3358 (99.7)	11 (0.3)	
	4A	55 (100.0)	0 (0.0)	
cn2	0	1932 (99.8)	3 (0.2)	0.717
	1	1757 (99.7)	5 (0.3)	
	2	908 (99.7)	3 (0.3)	
	3	122 (100.0)	0 (0.0)	
chir_type1	1	527 (100.0)	0 (0.0)	0.386
	2	2326 (100.0)	1 (0.0)	
	3	728 (99.7)	2 (0.3)	
	4	78 (100.0)	0 (0.0)	
	5	44 (100.0)	0 (0.0)	
	7	45 (100.0)	0 (0.0)	
	888	992 (100.0)	0 (0.0)	
lauren	1	2887 (99.9)	3 (0.1)	0.001
	2	661 (99.7)	2 (0.3)	
	3	126 (100.0)	0 (0.0)	
	4	204 (98.6)	3 (1.4)	
adj_nivo	0	3417 (99.7)	11 (0.3)	0.116
	1	345 (100.0)	0 (0.0)	
	8	992 (100.0)	0 (0.0)	
eus	0	2044 (99.6)	8 (0.4)	0.096
	1	2683 (99.9)	3 (0.1)	
pet_scan	0	220 (99.5)	1 (0.5)	1.000
	1	4440 (99.8)	10 (0.2)	
DR	0	2604 (99.8)	5 (0.2)	0.483
	1	150 (99.3)	1 (0.7)	
	8	1850 (99.7)	5 (0.3)	
tumregres	1	727 (100.0)	0 (0.0)	<0.001
	2	776 (100.0)	0 (0.0)	
	3	1810 (100.0)	0 (0.0)	
	4	213 (99.1)	2 (0.9)	
	88	992 (100.0)	0 (0.0)	

chir_rad1	0	3260 (100.0)	1 (0.0)	0.125
	1	325 (99.7)	1 (0.3)	
	2	3 (100.0)	0 (0.0)	
	88	992 (100.0)	0 (0.0)	
mort30	0	3683 (99.8)	9 (0.2)	<0.001
	1	74 (97.4)	2 (2.6)	
	88	992 (100.0)	0 (0.0)	
mort90	0	3583 (99.8)	7 (0.2)	<0.001
	1	173 (97.7)	4 (2.3)	
	88	992 (100.0)	0 (0.0)	
pt	0	727 (100.0)	0 (0.0)	0.473
	1A	133 (100.0)	0 (0.0)	
	1B	549 (99.8)	1 (0.2)	
	2	728 (100.0)	0 (0.0)	
	3	1551 (100.0)	0 (0.0)	
	4A	38 (100.0)	0 (0.0)	
	4B	7 (100.0)	0 (0.0)	
	888	992 (100.0)	0 (0.0)	
	yis	5 (100.0)	0 (0.0)	
CROSS_compleet	0	592 (99.5)	3 (0.5)	0.304
	1	4162 (99.8)	8 (0.2)	
RT_compleet	0	95 (97.9)	2 (2.1)	0.006
	1	4659 (99.8)	9 (0.2)	
chemo_compleet	0	530 (99.8)	1 (0.2)	1.000
	1	4224 (99.8)	10 (0.2)	
reden_geen_resectie	1	313 (100.0)	0 (0.0)	0.767
	2	353 (100.0)	0 (0.0)	
	3	85 (100.0)	0 (0.0)	
	4	87 (100.0)	0 (0.0)	
	5	39 (100.0)	0 (0.0)	
	888	3762 (99.7)	11 (0.3)	
wk_eind_crt_resectie	Mean (SD)	10.9 (5.3)	9.4 (6.2)	0.336
chir	0	992 (100.0)	0 (0.0)	0.183
	1	3762 (99.7)	11 (0.3)	

Missing data analysis: pt		Not missing	Missing	p
leeft	Mean (SD)	65.9 (8.7)	64.2 (8.2)	0.254
gesl	1	3973 (99.3)	30 (0.7)	0.660
	2	758 (99.5)	4 (0.5)	
cci_cat	0	2402 (99.3)	16 (0.7)	0.994
	1	1450 (99.3)	10 (0.7)	
	2	717 (99.3)	5 (0.7)	
incjr	Mean (SD)	2018.4 (2.3)	2018.1 (2.0)	0.460
perf_stat	0	2487 (99.2)	19 (0.8)	0.877
	1	1685 (99.4)	11 (0.6)	
	2	137 (100.0)	0 (0.0)	
	3	5 (100.0)	0 (0.0)	
	4	1 (100.0)	0 (0.0)	
topo_sublok	C153	1 (100.0)	0 (0.0)	0.076
	C154	128 (99.2)	1 (0.8)	
	C155	4009 (99.2)	32 (0.8)	
	C158	17 (94.4)	1 (5.6)	
	C160	243 (100.0)	0 (0.0)	
	C167	282 (100.0)	0 (0.0)	
diffgrad	1	160 (98.8)	2 (1.2)	0.744
	2	2010 (99.5)	11 (0.5)	
	3	1779 (99.3)	12 (0.7)	
	4	1 (100.0)	0 (0.0)	
ct	1	11 (100.0)	0 (0.0)	<0.001
	1B	2 (66.7)	1 (33.3)	
	2	1318 (99.3)	9 (0.7)	
	3	3346 (99.3)	23 (0.7)	
	4A	54 (98.2)	1 (1.8)	
cn2	0	1922 (99.3)	13 (0.7)	0.700
	1	1747 (99.1)	15 (0.9)	
	2	905 (99.3)	6 (0.7)	
	3	122 (100.0)	0 (0.0)	
chir_type1	1	524 (99.4)	3 (0.6)	0.028
	2	2313 (99.4)	14 (0.6)	
	3	723 (99.0)	7 (1.0)	
	4	76 (97.4)	2 (2.6)	
	5	44 (100.0)	0 (0.0)	
	7	45 (100.0)	0 (0.0)	
	888	992 (100.0)	0 (0.0)	
lauren	1	2874 (99.4)	16 (0.6)	0.083
	2	658 (99.2)	5 (0.8)	
	3	126 (100.0)	0 (0.0)	
	4	203 (98.1)	4 (1.9)	
adj_nivo	0	3395 (99.0)	33 (1.0)	0.004
	1	344 (99.7)	1 (0.3)	
	8	992 (100.0)	0 (0.0)	
eus	0	2034 (99.1)	18 (0.9)	0.258
	1	2671 (99.4)	15 (0.6)	
pet_scan	0	219 (99.1)	2 (0.9)	1.000
	1	4419 (99.3)	31 (0.7)	
DR	0	2591 (99.3)	18 (0.7)	0.167
	1	148 (98.0)	3 (2.0)	
	8	1843 (99.4)	12 (0.6)	
tumregres	1	710 (97.7)	17 (2.3)	<0.001
	2	773 (99.6)	3 (0.4)	
	3	1809 (99.9)	1 (0.1)	
	4	214 (99.5)	1 (0.5)	
	88	992 (100.0)	0 (0.0)	

chir_rad1	0	3238 (99.3)	23 (0.7)	<0.001
	1	325 (99.7)	1 (0.3)	
	2	2 (66.7)	1 (33.3)	
	88	992 (100.0)	0 (0.0)	
mort30	0	3660 (99.1)	32 (0.9)	0.002
	1	74 (97.4)	2 (2.6)	
	88	992 (100.0)	0 (0.0)	
mort90	0	3560 (99.2)	30 (0.8)	0.001
	1	173 (97.7)	4 (2.3)	
	88	992 (100.0)	0 (0.0)	
pn	0	2168 (99.2)	18 (0.8)	<0.001
	1	865 (99.5)	4 (0.5)	
	1M	8 (88.9)	1 (11.1)	
	2	468 (99.8)	1 (0.2)	
	3	223 (100.0)	0 (0.0)	
	3A	4 (100.0)	0 (0.0)	
	3B	2 (100.0)	0 (0.0)	
	888	992 (100.0)	0 (0.0)	
CROSS_compleet	0	589 (99.0)	6 (1.0)	0.514
	1	4142 (99.3)	28 (0.7)	
RT_compleet	0	94 (96.9)	3 (3.1)	0.028
	1	4637 (99.3)	31 (0.7)	
chemo_compleet	0	528 (99.4)	3 (0.6)	0.874
	1	4203 (99.3)	31 (0.7)	
reden_geen_resectie	1	313 (100.0)	0 (0.0)	0.158
	2	353 (100.0)	0 (0.0)	
	3	85 (100.0)	0 (0.0)	
	4	87 (100.0)	0 (0.0)	
	5	39 (100.0)	0 (0.0)	
	888	3739 (99.1)	34 (0.9)	
wk_eind_crt_resectie	Mean (SD)	10.9 (5.3)	10.0 (4.3)	0.294
chir	0	992 (100.0)	0 (0.0)	0.005
	1	3739 (99.1)	34 (0.9)	

Missing data analysis: reden_geen_resectie		Not missing	Missing	p
leeft	Mean (SD)	65.8 (8.6)	69.8 (8.5)	<0.001
gesl	1	3913 (97.8)	90 (2.2)	0.116
	2	737 (96.7)	25 (3.3)	
cci_cat	0	2361 (97.6)	57 (2.4)	0.263
	1	1430 (97.9)	30 (2.1)	
	2	699 (96.8)	23 (3.2)	
incjr	Mean (SD)	2018.4 (2.3)	2019.5 (2.0)	<0.001
perf_stat	0	2467 (98.4)	39 (1.6)	<0.001
	1	1640 (96.7)	56 (3.3)	
	2	129 (94.2)	8 (5.8)	
	3	5 (100.0)	0 (0.0)	
	4	1 (100.0)	0 (0.0)	
topo_sublok	C153	1 (100.0)	0 (0.0)	0.004
	C154	119 (92.2)	10 (7.8)	
	C155	3950 (97.7)	91 (2.3)	
	C158	18 (100.0)	0 (0.0)	
	C160	239 (98.4)	4 (1.6)	
	C167	274 (97.2)	8 (2.8)	
diffgrad	1	154 (95.1)	8 (4.9)	0.057
	2	1985 (98.2)	36 (1.8)	
	3	1754 (97.9)	37 (2.1)	
	4	1 (100.0)	0 (0.0)	
ct	1	11 (100.0)	0 (0.0)	0.966
	1B	3 (100.0)	0 (0.0)	
	2	1293 (97.4)	34 (2.6)	
	3	3289 (97.6)	80 (2.4)	
	4A	54 (98.2)	1 (1.8)	
cn2	0	1889 (97.6)	46 (2.4)	0.914
	1	1722 (97.7)	40 (2.3)	
	2	890 (97.7)	21 (2.3)	
	3	118 (96.7)	4 (3.3)	
chir_type1	1	527 (100.0)	0 (0.0)	<0.001
	2	2327 (100.0)	0 (0.0)	
	3	730 (100.0)	0 (0.0)	
	4	78 (100.0)	0 (0.0)	
	5	44 (100.0)	0 (0.0)	
	7	45 (100.0)	0 (0.0)	
	888	877 (88.4)	115 (11.6)	
lauren	1	2830 (97.9)	60 (2.1)	0.712
	2	651 (98.2)	12 (1.8)	
	3	122 (96.8)	4 (3.2)	
	4	204 (98.6)	3 (1.4)	
adj_nivo	0	3428 (100.0)	0 (0.0)	<0.001
	1	345 (100.0)	0 (0.0)	
	8	877 (88.4)	115 (11.6)	
eus	0	1976 (96.3)	76 (3.7)	<0.001
	1	2648 (98.6)	38 (1.4)	
pet_scan	0	212 (95.9)	9 (4.1)	0.165
	1	4345 (97.6)	105 (2.4)	
DR	0	2542 (97.4)	67 (2.6)	<0.001
	1	138 (91.4)	13 (8.6)	
	8	1828 (98.5)	27 (1.5)	
tumregres	1	727 (100.0)	0 (0.0)	<0.001
	2	776 (100.0)	0 (0.0)	
	3	1810 (100.0)	0 (0.0)	
	4	215 (100.0)	0 (0.0)	
	88	877 (88.4)	115 (11.6)	

yptn	1	638 (100.0)	0 (0.0)	<0.001
	2	1523 (100.0)	0 (0.0)	
	3	87 (100.0)	0 (0.0)	
	4	1436 (100.0)	0 (0.0)	
	5	54 (100.0)	0 (0.0)	
	88	877 (88.4)	115 (11.6)	
chir_rad1	0	3261 (100.0)	0 (0.0)	<0.001
	1	326 (100.0)	0 (0.0)	
	2	3 (100.0)	0 (0.0)	
	88	877 (88.4)	115 (11.6)	
mort30	0	3692 (100.0)	0 (0.0)	<0.001
	1	76 (100.0)	0 (0.0)	
	88	877 (88.4)	115 (11.6)	
mort90	0	3590 (100.0)	0 (0.0)	<0.001
	1	177 (100.0)	0 (0.0)	
	88	877 (88.4)	115 (11.6)	
pt	0	727 (100.0)	0 (0.0)	<0.001
	1A	133 (100.0)	0 (0.0)	
	1B	550 (100.0)	0 (0.0)	
	2	728 (100.0)	0 (0.0)	
	3	1551 (100.0)	0 (0.0)	
	4A	38 (100.0)	0 (0.0)	
	4B	7 (100.0)	0 (0.0)	
	888	877 (88.4)	115 (11.6)	
	yis	5 (100.0)	0 (0.0)	
pn	0	2186 (100.0)	0 (0.0)	<0.001
	1	869 (100.0)	0 (0.0)	
	1M	9 (100.0)	0 (0.0)	
	2	469 (100.0)	0 (0.0)	
	3	223 (100.0)	0 (0.0)	
	3A	4 (100.0)	0 (0.0)	
	3B	2 (100.0)	0 (0.0)	
	888	877 (88.4)	115 (11.6)	
CROSS_compleet	0	576 (96.8)	19 (3.2)	0.237
	1	4074 (97.7)	96 (2.3)	
RT_compleet	0	96 (99.0)	1 (1.0)	0.574
	1	4554 (97.6)	114 (2.4)	
chemo_compleet	0	512 (96.4)	19 (3.6)	0.088
	1	4138 (97.7)	96 (2.3)	
chir	0	877 (88.4)	115 (11.6)	<0.001
	1	3773 (100.0)	0 (0.0)	

Missing data analysis: topo_sublok		Not missing	Missing	p
leeft	Mean (SD)	65.9 (8.7)	66.4 (8.6)	0.685
gesl	1	3958 (98.9)	45 (1.1)	0.525
	2	756 (99.2)	6 (0.8)	
cci_cat	0	2387 (98.7)	31 (1.3)	0.346
	1	1446 (99.0)	14 (1.0)	
	2	717 (99.3)	5 (0.7)	
incjr	Mean (SD)	2018.4 (2.3)	2017.3 (1.7)	<0.001
perf_stat	0	2482 (99.0)	24 (1.0)	0.954
	1	1676 (98.8)	20 (1.2)	
	2	136 (99.3)	1 (0.7)	
	3	5 (100.0)	0 (0.0)	
	4	1 (100.0)	0 (0.0)	
diffgrad	1	161 (99.4)	1 (0.6)	0.864
	2	2003 (99.1)	18 (0.9)	
	3	1771 (98.9)	20 (1.1)	
	4	1 (100.0)	0 (0.0)	
ct	1	11 (100.0)	0 (0.0)	0.729
	1B	3 (100.0)	0 (0.0)	
	2	1309 (98.6)	18 (1.4)	
	3	3336 (99.0)	33 (1.0)	
	4A	55 (100.0)	0 (0.0)	
cn2	0	1909 (98.7)	26 (1.3)	0.418
	1	1747 (99.1)	15 (0.9)	
	2	903 (99.1)	8 (0.9)	
	3	120 (98.4)	2 (1.6)	
lauren	1	2864 (99.1)	26 (0.9)	0.216
	2	652 (98.3)	11 (1.7)	
	3	126 (100.0)	0 (0.0)	
	4	205 (99.0)	2 (1.0)	
chir_type1	1	525 (99.6)	2 (0.4)	0.012
	2	2304 (99.0)	23 (1.0)	
	3	721 (98.8)	9 (1.2)	
	4	74 (94.9)	4 (5.1)	
	5	44 (100.0)	0 (0.0)	
	7	45 (100.0)	0 (0.0)	
	888	979 (98.7)	13 (1.3)	
adj_nivo	0	3390 (98.9)	38 (1.1)	0.115
	1	345 (100.0)	0 (0.0)	
	8	979 (98.7)	13 (1.3)	
eus	0	2028 (98.8)	24 (1.2)	0.688
	1	2659 (99.0)	27 (1.0)	
pet_scan	0	218 (98.6)	3 (1.4)	0.954
	1	4402 (98.9)	48 (1.1)	
DR	0	2592 (99.3)	17 (0.7)	0.003
	1	150 (99.3)	1 (0.7)	
	8	1823 (98.3)	32 (1.7)	
tumregres	1	714 (98.2)	13 (1.8)	0.084
	2	765 (98.6)	11 (1.4)	
	3	1798 (99.3)	12 (0.7)	
	4	214 (99.5)	1 (0.5)	
	88	979 (98.7)	13 (1.3)	
yptn	1	628 (98.4)	10 (1.6)	0.211
	2	1506 (98.9)	17 (1.1)	
	3	85 (97.7)	2 (2.3)	
	4	1428 (99.4)	8 (0.6)	
	5	53 (98.1)	1 (1.9)	
	88	979 (98.7)	13 (1.3)	

chir_rad1	0	3223 (98.8)	38 (1.2)	0.247
	1	326 (100.0)	0 (0.0)	
	2	3 (100.0)	0 (0.0)	
	88	979 (98.7)	13 (1.3)	
mort30	0	3655 (99.0)	37 (1.0)	0.689
	1	75 (98.7)	1 (1.3)	
	88	979 (98.7)	13 (1.3)	
mort90	0	3553 (99.0)	37 (1.0)	0.601
	1	176 (99.4)	1 (0.6)	
	88	979 (98.7)	13 (1.3)	
pt	0	714 (98.2)	13 (1.8)	0.170
	1A	130 (97.7)	3 (2.3)	
	1B	542 (98.5)	8 (1.5)	
	2	723 (99.3)	5 (0.7)	
	3	1542 (99.4)	9 (0.6)	
	4A	38 (100.0)	0 (0.0)	
	4B	7 (100.0)	0 (0.0)	
	888	979 (98.7)	13 (1.3)	
	yis	5 (100.0)	0 (0.0)	
pn	0	2158 (98.7)	28 (1.3)	0.680
	1	862 (99.2)	7 (0.8)	
	1M	9 (100.0)	0 (0.0)	
	2	467 (99.6)	2 (0.4)	
	3	222 (99.6)	1 (0.4)	
	3A	4 (100.0)	0 (0.0)	
	3B	2 (100.0)	0 (0.0)	
	888	979 (98.7)	13 (1.3)	
CROSS_compleet	0	589 (99.0)	6 (1.0)	1.000
	1	4125 (98.9)	45 (1.1)	
RT_compleet	0	97 (100.0)	0 (0.0)	0.592
	1	4617 (98.9)	51 (1.1)	
chemo_compleet	0	525 (98.9)	6 (1.1)	1.000
	1	4189 (98.9)	45 (1.1)	
reden_geen_resectie	1	309 (98.7)	4 (1.3)	0.640
	2	348 (98.6)	5 (1.4)	
	3	83 (97.6)	2 (2.4)	
	4	87 (100.0)	0 (0.0)	
	5	39 (100.0)	0 (0.0)	
	888	3735 (99.0)	38 (1.0)	
wk_eind_crt_resectie	Mean (SD)	10.9 (5.3)	10.1 (3.3)	0.324
chir	0	979 (98.7)	13 (1.3)	0.514
	1	3735 (99.0)	38 (1.0)	

Missing data analysis: tumregres		Not missing	Missing	p
leeft	Mean (SD)	65.9 (8.7)	66.0 (8.5)	0.916
gesl	1	3801 (95.0)	202 (5.0)	0.552
	2	719 (94.4)	43 (5.6)	
cci_cat	0	2335 (96.6)	83 (3.4)	0.819
	1	1405 (96.2)	55 (3.8)	
	2	698 (96.7)	24 (3.3)	
incjr	Mean (SD)	2018.5 (2.3)	2017.9 (2.2)	<0.001
perf_stat	0	2439 (97.3)	67 (2.7)	0.981
	1	1648 (97.2)	48 (2.8)	
	2	134 (97.8)	3 (2.2)	
	3	5 (100.0)	0 (0.0)	
	4	1 (100.0)	0 (0.0)	
topo_sublok	C153	1 (100.0)	0 (0.0)	<0.001
	C154	124 (96.1)	5 (3.9)	
	C155	3851 (95.3)	190 (4.7)	
	C158	16 (88.9)	2 (11.1)	
	C160	201 (82.7)	42 (17.3)	
	C167	277 (98.2)	5 (1.8)	
diffgrad	1	155 (95.7)	7 (4.3)	0.906
	2	1907 (94.4)	114 (5.6)	
	3	1692 (94.5)	99 (5.5)	
	4	1 (100.0)	0 (0.0)	
ct	1	10 (90.9)	1 (9.1)	0.776
	1B	3 (100.0)	0 (0.0)	
	2	1259 (94.9)	68 (5.1)	
	3	3194 (94.8)	175 (5.2)	
	4A	54 (98.2)	1 (1.8)	
cn2	0	1845 (95.3)	90 (4.7)	0.674
	1	1668 (94.7)	94 (5.3)	
	2	861 (94.5)	50 (5.5)	
	3	117 (95.9)	5 (4.1)	
chir_type1	1	414 (78.6)	113 (21.4)	<0.001
	2	2283 (98.1)	44 (1.9)	
	3	712 (97.5)	18 (2.5)	
	4	33 (42.3)	45 (57.7)	
	5	44 (100.0)	0 (0.0)	
	7	42 (93.3)	3 (6.7)	
	888	992 (100.0)	0 (0.0)	
lauren	1	2738 (94.7)	152 (5.3)	0.092
	2	634 (95.6)	29 (4.4)	
	3	115 (91.3)	11 (8.7)	
	4	191 (92.3)	16 (7.7)	
adj_nivo	0	3210 (93.6)	218 (6.4)	<0.001
	1	318 (92.2)	27 (7.8)	
	8	992 (100.0)	0 (0.0)	
eus	0	1989 (96.9)	63 (3.1)	<0.001
	1	2524 (94.0)	162 (6.0)	
pet_scan	0	192 (86.9)	29 (13.1)	<0.001
	1	4322 (97.1)	128 (2.9)	
DR	0	2560 (98.1)	49 (1.9)	<0.001
	1	149 (98.7)	2 (1.3)	
	8	1730 (93.3)	125 (6.7)	
yptn	1	621 (97.3)	17 (2.7)	<0.001
	2	1415 (92.9)	108 (7.1)	
	3	85 (97.7)	2 (2.3)	
	4	1337 (93.1)	99 (6.9)	
	5	47 (87.0)	7 (13.0)	

	88	992 (100.0)	0 (0.0)	
chir_rad1	0	3173 (97.3)	88 (2.7)	<0.001
	1	319 (97.9)	7 (2.1)	
	2	3 (100.0)	0 (0.0)	
	88	992 (100.0)	0 (0.0)	
mort30	0	3455 (93.6)	237 (6.4)	<0.001
	1	69 (90.8)	7 (9.2)	
	88	992 (100.0)	0 (0.0)	
mort90	0	3362 (93.6)	228 (6.4)	<0.001
	1	161 (91.0)	16 (9.0)	
	88	992 (100.0)	0 (0.0)	
pt	0	708 (97.4)	19 (2.6)	<0.001
	1A	120 (90.2)	13 (9.8)	
	1B	515 (93.6)	35 (6.4)	
	2	683 (93.8)	45 (6.2)	
	3	1434 (92.5)	117 (7.5)	
	4A	35 (92.1)	3 (7.9)	
	4B	6 (85.7)	1 (14.3)	
	888	992 (100.0)	0 (0.0)	
	yis	5 (100.0)	0 (0.0)	
pn	0	2058 (94.1)	128 (5.9)	<0.001
	1	813 (93.6)	56 (6.4)	
	1M	9 (100.0)	0 (0.0)	
	2	438 (93.4)	31 (6.6)	
	3	202 (90.6)	21 (9.4)	
	3A	4 (100.0)	0 (0.0)	
	3B	2 (100.0)	0 (0.0)	
	888	992 (100.0)	0 (0.0)	
CROSS_compleet	0	572 (96.1)	23 (3.9)	0.159
	1	3948 (94.7)	222 (5.3)	
RT_compleet	0	92 (94.8)	5 (5.2)	1.000
	1	4428 (94.9)	240 (5.1)	
chemo_compleet	0	513 (96.6)	18 (3.4)	0.067
	1	4007 (94.6)	227 (5.4)	
reden_geen_resectie	1	313 (100.0)	0 (0.0)	<0.001
	2	353 (100.0)	0 (0.0)	
	3	85 (100.0)	0 (0.0)	
	4	87 (100.0)	0 (0.0)	
	5	39 (100.0)	0 (0.0)	
	888	3528 (93.5)	245 (6.5)	
wk_eind_crt_resectie	Mean (SD)	11.0 (5.4)	10.1 (4.2)	0.007
chir	0	992 (100.0)	0 (0.0)	<0.001
	1	3528 (93.5)	245 (6.5)	

Missing data analysis: wk_eind_crt_resectie		Not missing	Missing	p
leeft	Mean (SD)	65.3 (8.5)	68.3 (8.8)	<0.001
gesl	1	3189 (79.7)	814 (20.3)	0.038
	2	581 (76.2)	181 (23.8)	
cci_cat	0	1941 (80.3)	477 (19.7)	<0.001
	1	1148 (78.6)	312 (21.4)	
	2	527 (73.0)	195 (27.0)	
incjr	Mean (SD)	2018.2 (2.3)	2019.2 (2.2)	<0.001
perf_stat	0	2073 (82.7)	433 (17.3)	<0.001
	1	1249 (73.6)	447 (26.4)	
	2	81 (59.1)	56 (40.9)	
	3	3 (60.0)	2 (40.0)	
	4	1 (100.0)	0 (0.0)	
topo_sublok	C153	1 (100.0)	0 (0.0)	0.074
	C154	96 (74.4)	33 (25.6)	
	C155	3207 (79.4)	834 (20.6)	
	C158	15 (83.3)	3 (16.7)	
	C160	204 (84.0)	39 (16.0)	
	C167	209 (74.1)	73 (25.9)	
diffgrad	1	111 (68.5)	51 (31.5)	<0.001
	2	1688 (83.5)	333 (16.5)	
	3	1442 (80.5)	349 (19.5)	
	4	0 (0.0)	1 (100.0)	
ct	1	10 (90.9)	1 (9.1)	0.408
	1B	2 (66.7)	1 (33.3)	
	2	1070 (80.6)	257 (19.4)	
	3	2646 (78.5)	723 (21.5)	
	4A	42 (76.4)	13 (23.6)	
cn2	0	1571 (81.2)	364 (18.8)	0.005
	1	1391 (78.9)	371 (21.1)	
	2	696 (76.4)	215 (23.6)	
	3	88 (72.1)	34 (27.9)	
chir_type1	1	527 (100.0)	0 (0.0)	<0.001
	2	2325 (99.9)	2 (0.1)	
	3	729 (99.9)	1 (0.1)	
	4	78 (100.0)	0 (0.0)	
	5	44 (100.0)	0 (0.0)	
	7	45 (100.0)	0 (0.0)	
	888	0 (0.0)	992 (100.0)	
lauren	1	2416 (83.6)	474 (16.4)	<0.001
	2	494 (74.5)	169 (25.5)	
	3	95 (75.4)	31 (24.6)	
	4	182 (87.9)	25 (12.1)	
adj_nivo	0	3425 (99.9)	3 (0.1)	<0.001
	1	345 (100.0)	0 (0.0)	
	8	0 (0.0)	992 (100.0)	
eus	0	1596 (77.8)	456 (22.2)	0.072
	1	2148 (80.0)	538 (20.0)	
pet_scan	0	177 (80.1)	44 (19.9)	0.670
	1	3500 (78.7)	950 (21.3)	
DR	0	1946 (74.6)	663 (25.4)	<0.001
	1	90 (59.6)	61 (40.4)	
	8	1609 (86.7)	246 (13.3)	
tumregres	1	727 (100.0)	0 (0.0)	<0.001
	2	774 (99.7)	2 (0.3)	
	3	1810 (100.0)	0 (0.0)	
	4	214 (99.5)	1 (0.5)	
	88	0 (0.0)	992 (100.0)	

yptn	1	638 (100.0)	0 (0.0)	<0.001
	2	1521 (99.9)	2 (0.1)	
	3	87 (100.0)	0 (0.0)	
	4	1435 (99.9)	1 (0.1)	
	5	54 (100.0)	0 (0.0)	
	88	0 (0.0)	992 (100.0)	
chir_rad1	0	3258 (99.9)	3 (0.1)	<0.001
	1	326 (100.0)	0 (0.0)	
	2	3 (100.0)	0 (0.0)	
	88	0 (0.0)	992 (100.0)	
mort30	0	3689 (99.9)	3 (0.1)	<0.001
	1	76 (100.0)	0 (0.0)	
	88	0 (0.0)	992 (100.0)	
mort90	0	3588 (99.9)	2 (0.1)	<0.001
	1	176 (99.4)	1 (0.6)	
	88	0 (0.0)	992 (100.0)	
pt	0	727 (100.0)	0 (0.0)	<0.001
	1A	133 (100.0)	0 (0.0)	
	1B	549 (99.8)	1 (0.2)	
	2	727 (99.9)	1 (0.1)	
	3	1550 (99.9)	1 (0.1)	
	4A	38 (100.0)	0 (0.0)	
	4B	7 (100.0)	0 (0.0)	
	888	0 (0.0)	992 (100.0)	
	yis	5 (100.0)	0 (0.0)	
pn	0	2184 (99.9)	2 (0.1)	<0.001
	1	868 (99.9)	1 (0.1)	
	1M	9 (100.0)	0 (0.0)	
	2	469 (100.0)	0 (0.0)	
	3	223 (100.0)	0 (0.0)	
	3A	4 (100.0)	0 (0.0)	
	3B	2 (100.0)	0 (0.0)	
	888	0 (0.0)	992 (100.0)	
CROSS_compleet	0	420 (70.6)	175 (29.4)	<0.001
	1	3350 (80.3)	820 (19.7)	
RT_compleet	0	65 (67.0)	32 (33.0)	0.005
	1	3705 (79.4)	963 (20.6)	
chemo_compleet	0	364 (68.5)	167 (31.5)	<0.001
	1	3406 (80.4)	828 (19.6)	
reden_geen_resectie	1	0 (0.0)	313 (100.0)	<0.001
	2	0 (0.0)	353 (100.0)	
	3	0 (0.0)	85 (100.0)	
	4	0 (0.0)	87 (100.0)	
	5	0 (0.0)	39 (100.0)	
	888	3770 (99.9)	3 (0.1)	
chir	0	0 (0.0)	992 (100.0)	<0.001
	1	3770 (99.9)	3 (0.1)	

Missing data analysis: yptn		Not missing	Missing	p
leeft	Mean (SD)	65.9 (8.7)	64.2 (8.1)	0.245
gesl	1	3972 (99.2)	31 (0.8)	0.612
	2	758 (99.5)	4 (0.5)	
cci_cat	0	2402 (99.3)	16 (0.7)	0.946
	1	1449 (99.2)	11 (0.8)	
	2	717 (99.3)	5 (0.7)	
incjr	Mean (SD)	2018.4 (2.3)	2018.1 (2.0)	0.325
perf_stat	0	2486 (99.2)	20 (0.8)	0.847
	1	1685 (99.4)	11 (0.6)	
	2	137 (100.0)	0 (0.0)	
	3	5 (100.0)	0 (0.0)	
	4	1 (100.0)	0 (0.0)	
topo_sublok	C153	1 (100.0)	0 (0.0)	0.078
	C154	128 (99.2)	1 (0.8)	
	C155	4008 (99.2)	33 (0.8)	
	C158	17 (94.4)	1 (5.6)	
	C160	243 (100.0)	0 (0.0)	
	C167	282 (100.0)	0 (0.0)	
diffgrad	1	160 (98.8)	2 (1.2)	0.809
	2	2009 (99.4)	12 (0.6)	
	3	1779 (99.3)	12 (0.7)	
	4	1 (100.0)	0 (0.0)	
ct	1	11 (100.0)	0 (0.0)	<0.001
	1B	2 (66.7)	1 (33.3)	
	2	1318 (99.3)	9 (0.7)	
	3	3345 (99.3)	24 (0.7)	
	4A	54 (98.2)	1 (1.8)	
cn2	0	1921 (99.3)	14 (0.7)	0.730
	1	1747 (99.1)	15 (0.9)	
	2	905 (99.3)	6 (0.7)	
	3	122 (100.0)	0 (0.0)	
chir_type1	1	524 (99.4)	3 (0.6)	0.018
	2	2313 (99.4)	14 (0.6)	
	3	722 (98.9)	8 (1.1)	
	4	76 (97.4)	2 (2.6)	
	5	44 (100.0)	0 (0.0)	
	7	45 (100.0)	0 (0.0)	
	888	992 (100.0)	0 (0.0)	
lauren	1	2874 (99.4)	16 (0.6)	0.083
	2	658 (99.2)	5 (0.8)	
	3	126 (100.0)	0 (0.0)	
	4	203 (98.1)	4 (1.9)	
adj_nivo	0	3394 (99.0)	34 (1.0)	0.003
	1	344 (99.7)	1 (0.3)	
	8	992 (100.0)	0 (0.0)	
eus	0	2033 (99.1)	19 (0.9)	0.190
	1	2671 (99.4)	15 (0.6)	
pet_scan	0	219 (99.1)	2 (0.9)	1.000
	1	4418 (99.3)	32 (0.7)	
DR	0	2591 (99.3)	18 (0.7)	0.189
	1	148 (98.0)	3 (2.0)	
	8	1842 (99.3)	13 (0.7)	
tumregres	1	710 (97.7)	17 (2.3)	<0.001
	2	773 (99.6)	3 (0.4)	
	3	1809 (99.9)	1 (0.1)	
	4	213 (99.1)	2 (0.9)	
	88	992 (100.0)	0 (0.0)	

chir_rad1	0	3237 (99.3)	24 (0.7)	<0.001
	1	325 (99.7)	1 (0.3)	
	2	2 (66.7)	1 (33.3)	
	88	992 (100.0)	0 (0.0)	
mort30	0	3659 (99.1)	33 (0.9)	0.002
	1	74 (97.4)	2 (2.6)	
	88	992 (100.0)	0 (0.0)	
mort90	0	3559 (99.1)	31 (0.9)	0.001
	1	173 (97.7)	4 (2.3)	
	88	992 (100.0)	0 (0.0)	
pt	0	727 (100.0)	0 (0.0)	0.473
	1A	133 (100.0)	0 (0.0)	
	1B	549 (99.8)	1 (0.2)	
	2	728 (100.0)	0 (0.0)	
	3	1551 (100.0)	0 (0.0)	
	4A	38 (100.0)	0 (0.0)	
	4B	7 (100.0)	0 (0.0)	
	888	992 (100.0)	0 (0.0)	
	yis	5 (100.0)	0 (0.0)	
pn	0	2168 (99.2)	18 (0.8)	<0.001
	1	865 (99.5)	4 (0.5)	
	1M	8 (88.9)	1 (11.1)	
	2	468 (99.8)	1 (0.2)	
	3	223 (100.0)	0 (0.0)	
	3A	4 (100.0)	0 (0.0)	
	3B	2 (100.0)	0 (0.0)	
	888	992 (100.0)	0 (0.0)	
CROSS_compleet	0	589 (99.0)	6 (1.0)	0.562
	1	4141 (99.3)	29 (0.7)	
RT_compleet	0	94 (96.9)	3 (3.1)	0.032
	1	4636 (99.3)	32 (0.7)	
chemo_compleet	0	528 (99.4)	3 (0.6)	0.829
	1	4202 (99.2)	32 (0.8)	
reden_geen_resectie	1	313 (100.0)	0 (0.0)	0.146
	2	353 (100.0)	0 (0.0)	
	3	85 (100.0)	0 (0.0)	
	4	87 (100.0)	0 (0.0)	
	5	39 (100.0)	0 (0.0)	
	888	3738 (99.1)	35 (0.9)	
wk_eind_crt_resectie	Mean (SD)	10.9 (5.3)	9.9 (4.2)	0.257
chir	0	992 (100.0)	0 (0.0)	0.005
	1	3738 (99.1)	35 (0.9)	
