# Supplementary material for: Real-world outcomes of the CROSS regimen in patients with resectable esophageal or gastro-esophageal junction adenocarcinoma: a nationwide cohort study in the Netherlands
Source: eClinicalMedicine. 2025 Jan 22;80:103067. doi: 10.1016/j.eclinm.2024.103067 (PMC11795631; doi:10.1016/j.eclinm.2024.103067)
Supplement: Supplementary Figure S1 — Overall survival (OS) of pre-SANO cohort (1A) and Not SANO centers since start of neoadjuvant chemoradiotherapy. [file mmc3.pdf]

1A

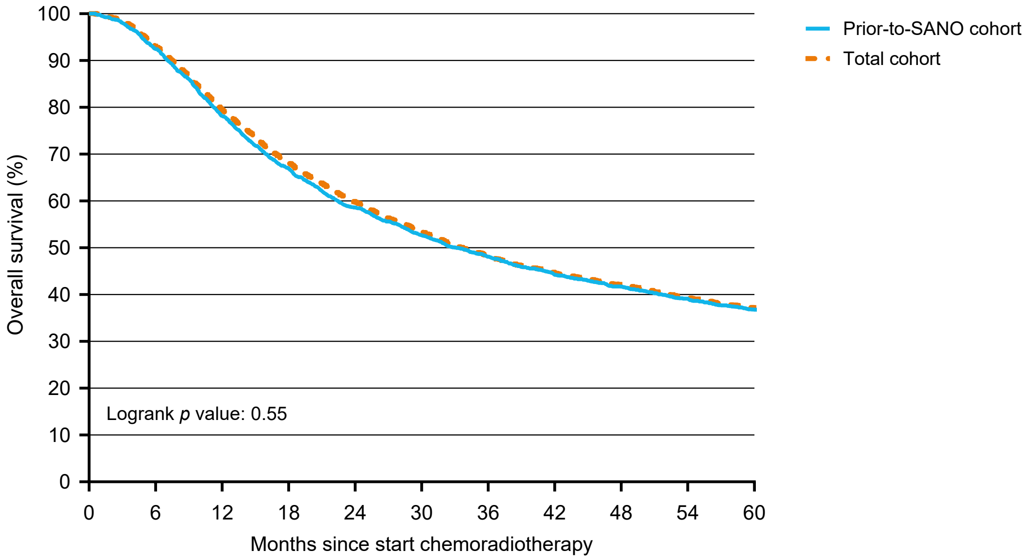

**Numbers at risk**											
Prior-to-SANO cohort	1854	1717	1450	1244	1088	978	893	821	773	726	682
Total cohort	4761	4433	3771	3044	2493	2037	1712	1458	1271	1079	901

1B

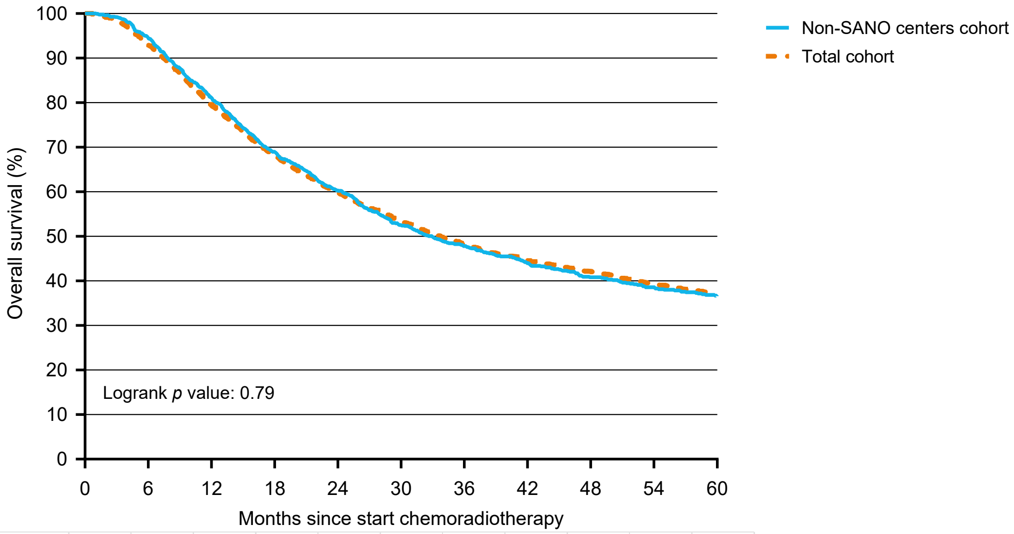

**Numbers at risk**											
Non-SANO centers cohort	1254	1188	1014	806	657	524	444	373	323	283	244
Total cohort	4761	4433	3771	3044	2493	2037	1712	1458	1271	1079	901

Supplementary figure S1. *Overall survival (OS) of pre-SANO cohort (1A) and Not SANO centers since start of neoadjuvant chemoradiotherapy*
